# Making Nations Great Again: National Narcissism and the Left, the Right, and the Extreme in the European Context

**DOI:** 10.5334/irsp.844

**Published:** 2024-02-12

**Authors:** Marina Maglić, Tomislav Pavlović, Renata Franc

**Affiliations:** 1 Ivo Pilar Institute of Social Sciences, Croatia

**Keywords:** collective narcissism, national narcissism, political extremity, political ideology, political orientation, Europe, cross-national analyses

## Abstract

Considering current world politics, investigating people’s tendency to believe in the greatness of their nation contingent on external validation seems ever so relevant. Thus, we examined the relationship between the direction and extremity of political orientation and national narcissism (NN) on European quota-representative samples (*N* = 15,882). Although the relationships between ideological extremity and NN were established, they were much weaker than the positive relationship between political orientation and NN. Testing for model invariance across Western and Eastern European samples suggested differences in the predictive strength of political orientation on NN, with a weaker association in Eastern Europe. Further analyses, including a quadratic examination of political ideology’s relation to NN, supported the rigidity-of-the-right hypothesis, highlighting the stronger presence of NN among right-leaning individuals. This pattern persisted despite varying European socio-cultural backgrounds, suggesting a transcultural aspect of NN within the political right spectrum. Our research underscores the importance of considering socio-political context when assessing the interplay between political ideology and NN, setting a starting point for further, more nuanced research.

## Introduction

Identity dynamics, from individual, ethnic, national to supranational level, have a profound impact on the political sphere. Namely, the quest for recognition of one’s identity, especially one’s national superiority and entitlement, has emerged as a key driving force in contemporary world politics. Collective narcissism—extrapolated from individual narcissism to a broader social identity context—articulates an inflated belief in a group’s exceptionality and entitlement that hinges on external affirmation (Golec de Zavala et al., 2009; Golec de Zavala, Dyduch-Hazar, & Lantos, 2019, Golec de Zavala et al., 2016).

### Collective National Narcissism

This form of positive ingroup identification can be related to any social group (e.g., Golec de Zavala, Cichocka, & Bilewicz, 2013; Marchlewska et al., 2019), which is reflected in its measure devoid of specific national or cultural context (Cichocka, 2016). Collective narcissism with respect to one’s national identity, that is, national narcissism (NN) is akin to nationalism (and blind patriotism) in its promotion of ingroup superiority and entitlement but diverges in its fundamental motivations. Over and above this conceptual overlap of the constructs, nationalism is orientated first and foremost toward asserting national dominance, while NN is primarily driven by a defensive need for ingroup recognition, whether through group aggrandizement or asserting dominance (Cichocka & Cislak, 2020; Golec de Zavala, 2018; Golec de Zavala et al., 2009, 2019).

Furthermore, NN is distinct from basic positive ingroup identification, although both comprise a positive ingroup evaluation (Cichocka, 2016). Namely, once this overlap is accounted for, national identification is related to constructive, tolerant intergroup attitudes, while NN is related to negative attitudes and hostility toward outgroups (see Cichocka, 2016; Cichocka & Cislak, 2020, for review). National narcissism (but not national identification) has also been related to conspiratorial thinking regarding malevolent plotting of specific outgroups which are perceived as threatening to the ingroup, as well as with a general tendency towards conspiratorial thinking (Golec de Zavala, 2020; Golec de Zavala & Keenan, 2020; Sternisko et al., 2021). Moreover, recent research (reviewed by Golec de Zavala & Keenan, 2020; for a meta-analysis, see Forgas & Lantos, 2019) highlights the role of NN in support of isolationist, anti-liberal populistic movements (e.g., supporters of Brexit and Polexit, Donald Trump supporters, supporters of the Fidesz party and Viktor Orbán; supporters of PiS and ONR in Poland).

Such adverse social outcomes of NN are reliably reported and seem to stem from a perceived lack of ingroup recognition (see Cichocka, 2016, and Golec de Zavala & Lantos, 2020, for reviews). Consistent with the two-factor models of narcissism as an individual characteristic that can be divided into vulnerable and grandiose components (Miller et al., 2011; Pincus et al., 2009), national narcissists tend to hold an exceptionally positive yet fragile opinion of their ingroup, undermined by constant doubts (see Golec de Zavala, 2011). These doubts lead to hypersensitivity to group-based criticism and motivate national narcissists to keep proving the superiority of their ingroup both to themselves and outgroups, which are perceived as insufficiently appreciative of the ingroup. This (consistent) lack of acceptable acknowledgment can lead to outgroup hostility and even violence (Golec de Zavala, 2011; Golec de Zavala et al., 2019) as ‘non-appreciative’ outgroups are seen as potential threats (Adorno, 1951; Lyons, Kenworthy, & Popan, 2010) to ingroup’s privileged status and positive image, regardless of when the threat occurred and whether it was real (Golec de Zavala, 2018; Golec de Zavala & Lantos, 2020). For instance, an extremely positive opinion regarding one’s ingroup is also an element of identity fusion (Swann & Buhrmester, 2015; Swann et al., 2012), recognized as an important factor of willingness to conduct suicide attacks against outgroups. Overall, there is a growing body of research on the detrimental consequences of this form of national identification (e.g., Golec de Zavala et al., 2009), although admittedly correlational in nature. However, research has not focused extensively on the antecedents, beyond exploring the relationships with other forms of national identification (e.g., Cichocka et al., 2023).

### Disentangling Political Ideology: The Rigidity-of-the-Right and the Ideological Extremity Hypotheses

We are profoundly social and political creatures, with politics affecting many aspects of our lives, from how much tax we pay, how educational and health care systems should be conceived, and which interpersonal relationships are recognized by law to immigration and environmental policies. Consequently, it is difficult to find domains utterly devoid of the influence of political ideology and its manifestations. In addition to having specific ideological beliefs and policy preferences, individuals generally have an overarching political orientation which can be assessed along a left-right or liberal-conservative dimension. This parsimonious ideological dimension has been ubiquitous in political science research worldwide (Huber & Inglehart, 1995; Jost, 2006; Wiesehomeier & Benoit, 2009). Indeed, it has been shown to predict various phenomena, from attitudes toward moral transgressors (Smith et al., 2019), prosociality (Osborne & Weiner, 2015; Van Lange et al., 2012), well-being (Napier & Jost, 2008), attributions of luck and success (Gromet, Hartson, & Sherman, 2015), to interpersonal relationships (Chopik & Motyl, 2016), to name a few. Regarding the relationship with different forms of national attachment, there is evidence that nationalism and patriotism are often higher among conservatives than liberals (e.g., Huddy & Khatib, 2007; Van der Toorn et al., 2014). On the other hand, basic national identity or identification is sometimes found to be equally endorsed by conservatives and liberals (e.g., Huddy & Khatib, 2007) as well as positively related to right, conservative orientation (e.g., Cichocka et al., 2016; Cislak, Wojcik, & Cichocka 2018; Van der Toorn et al., 2014).

However, the *rigidity-of-the-right hypothesis* and the *ideological extremity hypothesis* offer divergent perspectives on how political ideology relates to psychological traits and outcomes and, by extension, to NN.

#### Rigidity-of-the-right

Psychologists have long proposed the idea that ideological preferences might be rooted in differences in basic psychological values, dispositions, and needs, with the research of this line of thought indicating that conservatives and right-oriented individuals are more likely to resist change and justify and support inequality (Jost et al., 2003b), put greater emphasis on conformity and tradition over universalism and benevolence values (e.g., Caprara et al., 2017; Piurko, Schwartz, & Davidov, 2011; Thorisdottir et al., 2007), and evade consequentialist thinking (i.e., are prone to deontological moral judgments; Piazza & Sousa, 2014). The assumption about the existence of underlying psychological differences along the ideological lines is most prominently articulated within the *rigidity-of-the-right hypothesis*—the idea that a conservative political orientation is associated with psychological rigidity (Altemeyer, 1998; Jost, 2017; Stone, 1980; Tetlock, Bernzweig, & Gallant, 1985). Recent meta-analyses (Jost, 2017; Jost, Sterling, & Stern 2017; Van Hiel et al., 2016) re-evaluated the evidence in favor of this account, with the overall findings corroborating that conservatism was related to dogmatism, intolerance of ambiguity, and uncertainty avoidance.

This psychological profile suggests that right-wing individuals might be more prone to NN due to their predisposition toward upholding in-group superiority and resisting external influences that could undermine this perception. Indeed, evidence suggests that NN tends to be positively related to right-wing attitudes, beliefs, and orientations, such as right-wing authoritarianism, and social dominance orientation (e.g., Cichocka et al., 2017; Golec de Zavala, Guerra, & Simão, 2017, but see Golec de Zavala et al., 2009, who did not find evidence of associations on a Mexican sample in Study 5), conservative and right-leaning ideology (Bocian, Cichocka, & Wojciszke, 2021; Cichocka et al., 2016; Cislak et al., 2020; Górska et al., 2022; Sternisko et al., 2021; Verkuyten et al., 2022).

#### Ideological extremity

Other scholars advocate the *ideological extremity hypothesis*, positing that ideological extremity, whether political left or right, might stem from similar underlying psychological underpinnings with individuals on both sides of the political extremes being more cognitively rigid, dogmatic, intolerant, more overconfident, and feeling more superior about own beliefs than political moderates (Fernbach et al., 2013; Greenberg & Jonas, 2003; Toner et al., 2013; Van Prooijen & Krouwel, 2019). Indeed, it has been shown that both the extreme left and the extreme right derogate groups they perceive as dissimilar (Chambers, Schlenker, & Collisson, 2013; Wetherell, Brandt, & Reyna, 2013; for a joint discussion see Brandt et al., 2014), which seems to be mediated by the perceived violation of ingroup values (Wetherell et al., 2013). The difference lies in selecting groups, as the extreme right targets different ones (e.g., immigrants and LGBT individuals) than the extreme left (e.g., Christians and bankers). One of the clearest arguments supporting this hypothesis was provided by Van Prooijen, Krouwel, Boiten, and Eendebak (2015). They found that both the extreme left and the extreme right exhibit stronger negative emotions about the current political system than moderates, accompanied by stronger socio-economic fear and derogation of out-groups. Furthermore, both extremes seem to be more confident in the simplicity of solutions to political problems and feel more confident in their knowledge of the problem regardless of their actual knowledge, which was shown in the context of the immigration crisis in Europe in 2016 (Van Prooijen, Krouwel, & Emmer, 2018). Also, the two extremes seem more inclined to political distrust and Euroscepticism, a policy that may be perceived as threatening to one’s national identity, than political moderates (Kutiyski, Krouwel, & van Prooijen, 2021). However, these tendencies are generally more characteristic of individuals on the extreme right than those on the extreme left (Kutiyski et al., 2021). Listed findings align with the recent studies emphasizing the relevance of left-wing authoritarianism (Conway III et al., 2021; Costello et al., 2022).

Such extremity could relate to NN in the sense that both left and right extremists may adopt narcissistic views of their nation to reinforce their ideological purity and to delineate clear boundaries against perceived outgroups.

#### Socio-political contexts

Overall, the distinction along the left-right or liberal-conservative dimension was relatively stable, enduring, and relevant (Bobbio, 1996; Corbetta, Cavazza, & Roccato, 2009; Jost, Federico, & Napier, 2009). Nevertheless, there may be some heterogeneity in the underlying meaning of the left and right continuum across countries and political contexts (Greenberg & Jonas, 2003; Huber & Inglehart, 1995). Evidence from cross-cultural research suggests that the political right and left, or conservatives and liberals, to an extent hold different values (and aim to achieve different goals in different ways) in different political and cultural contexts (e.g., Aspelund, Lindeman, & Verkasalo, 2013; Piurko et al., 2011; Thorisdottir et al., 2007). In sum, it seems that in Western established democracies, the meaning of the left-right dimension is rather coherent, whereas, in socialist countries and ones with a history of the socialist or communist regime, it is inconsistent (being reversed or indistinguishable, e.g., Barni, Vieno & Roccato, 2016; Caprara et al., 2017; Malka et al., 2014). A tentative explanation may be that in the latter countries, individuals with a dispositional tendency towards security, stability, and order may be inclined to left-wing ideologies consistent with belief systems that dominated for most of past centuries, in addition to the experience of suppression of opposition in those regimes.

We must note that the *ideological extremity* and *rigidity-of-the-right* hypotheses are not necessarily mutually exclusive. As Jost, Glaser, Kruglanski, and Sulloway (2003a, 383) pointed out, ‘rigidity of the left can and does occur, but it is less common than the rigidity of the right.’ Although the data in their initial meta-analysis (2003a) did not allow for a comprehensive meta-analytic test for the extremity (quadratic) effect, seven out of 13 individual studies that allowed for a direct test between the two hypotheses indicated a linear relationship between conservatism and uncertainty/threat avoidance. However, the remaining six studies showed both linear and quadratic effects, thus providing some evidence for both hypotheses. In subsequent research, Jost et al. (2007) found no evidence that uncertainty and threat management are only associated with ideological extremism or extreme conservatism. In their cross-cultural study, Thorisdottir et al. (2007) detected a positive quadratic trend (especially in Western Europe), indicating that ideological extremity was generally associated with openness to experience. However, no such effect was observed regarding the need for order, rule-following, and security. Furthermore, Van Prooijen and colleagues detected both linear and quadratic relationships between political orientation and dogmatic intolerance over trivial issues as well as over political issues (Van Prooijen & Krouwel, 2017), and derogation of immigrants and different societal groups (Van Prooijen et al., 2015). Thus, they provided support for both hypotheses, that is, for the idea of stronger rigidity on the right of the political spectrum, but also for the notion that the strength or extremity of ideological beliefs is relevant above and beyond political orientation (see also Van Prooijen & Kuijper, 2020).

It is worth noting that the notion that both, left and right, ideological extremity share similar underlying psychology accords with theories of radicalization and extremism (e.g., Kruglanski et al., 2014, Kruglanski et al., 2017; McCauley & Moskalenko, 2008, 2017; see also Greenberg & Jonas, 2003). Indeed, these dynamics can manifest at the national level, with political extremism, both right and left, shown to be related to extreme nationalism, often preceded by feelings of injustice and humiliation, and accompanied by perceptions of threat (Midlarsky, 2011).

In sum, the ideological extremity hypothesis may be viewed as an extension of the rigidity of the right hypothesis that has the potential to account for atrocities caused by the extreme left and right regimes during the previous century. The keyword here is *potential*—despite the robustness of evidence they provided, Van Prooijen et al. (2015) raised doubts if these quadratic relationships can be generalized on all potentially relevant characteristics within the framework of psychological rigidity research and in every culture. Thus, an obvious need exists for further research on how the left and right differ and how both extremes differ from moderates and one another.

Furthermore, as is the case in many fields of psychological research, most findings regarding political ideology and NN are based on samples from predominately capitalist WEIRD (Western, Educated, Industrialized, Rich, and Democratic) countries (Henrich, Heine & Norenzayan, 2010). This represents a relevant limitation in the knowledge base as the basic presumptions underlying the political left and right may depend on the historical legacy (see Thorisdottir et al., 2007), while NN seems to depend on the contextual characteristics such as the level of globalization (Cichocka et al., 2023). Moreover, while there have been some noted efforts in cross-cultural research regarding political orientation, as previously mentioned, replications and a broader corpus of research in the context of countries with a history of movements and governments combining left-wing extremism and radicalism, nationalism, and NN are lacking.

### Linking Political Ideology and Extremity to National Narcissism Across Europe

The anticipated variations between Western and Eastern European countries in the dynamics of the relationship of political ideology and NN can be traced back to their distinct historical and political trajectories.

Namely, the inconsistency in the meaning of the left-right dimension Eastern European countries suggests that NN in Eastern European contexts might not align neatly with right-wing ideologies, as it does in the West, and could manifest across the political spectrum as a function of historical context rather than ideology alone. In Western Europe, where democratic institutions and norms have had a longer time to root and stabilize, right-wing ideologies may align with NN through the defense and promotion of national pride and identity within an established and secure system. In contrast, in Eastern European countries, where socialist legacies may still exert influence, left-wing ideologies could also be connected to NN, albeit for different reasons. Here, the post-socialist identity transformation and the struggle for a cohesive national narrative post-EU accession may lead to an association between left-wing beliefs and NN, as people search for a stable identity anchor in a rapidly changing political landscape (see for example Ekman & Linde, 2005; Howard, 2002; Sztompka, 1996, 2000;).

Moreover, the potential for left-wing authoritarianism in both Western and Eastern Europe suggests that NN could be a feature of extremism more generally, rather than being exclusive to the right. The need to defend the in-group and maintain its superiority could be a common thread among all forms of political extremism, thereby linking NN to both ideological rigidity and extremity, transcending traditional political divides.

Additionally, the relationship between political ideology and NN is further complicated by the dynamics within the European Union (EU). Euroscepticism, which is prevalent in varying degrees across the continent, often converges with NN as it emphasizes national exclusivity and distinctiveness over supranational identity (Hooghe & Marks, 2009). In Western Europe, Euroscepticism is associated with both the extreme right and left of the political spectrum (De Vries & Edwards, 2009; Kutiyski et al., 2021). Yet individuals on the extreme right seem to be more inclined to this view than those on the extreme left, aligning with NN through a focus on national autonomy and resistance to external influence (Kutiyski et al., 2021). Since there is evidence that extremism reduces uncertainty in new democracies (see Ezrow, Homola, & Tavits, 2014), in Eastern European countries, Euroscepticism may cross traditional left-right boundaries, often emerging from a sense of economic or cultural threat, thus linking to NN through perceived challenges to national esteem.

Moreover, the rise of populist movements across Europe has often been tied to NN, as these movements tend to invoke nationalistic sentiments that can serve as a catalyst for narcissistic expressions of national identity (Golec de Zavala et al., 2019; Lantos & Forgas, 2021; Marchlewska et al., 2018). Yet this relationship remains unclear and may vary across different sociopolitical contexts (see Cichocka et al., 2023).

To understand these diverse relationships fully, cross-cultural research is essential. Comparative studies, such as the one by Norris and Inglehart (2009), have provided insights into the complex ways in which political ideologies are shaped by and, in turn, shape national identity and sentiments of superiority or entitlement. Furthermore, studies on the political psychology of European integration offer a nuanced look at how political ideologies intersect with national identity within the context of the EU (Fligstein, Polyakova, & Sandholtz, 2012; Hooghe & Marks, 2009).

To date, the relationship between political ideology and NN was predominantly explored in Polish, British, German, Dutch, and US samples (Bocian et al., 2021; Cichocka et al., 2016; Cislak et al., 2020; Górska et al., 2022; Sternisko et al., 2021; Verkuyten et al., 2022). To our knowledge, political ideology and ideological extremity were investigated alongside NN only in a recent study in the USA (Golec de Zavala & Federico, 2018). Although a positive association (somewhat lower compared to the aforementioned studies) between NN and political conservativism was detected, authors found no evidence of its association with ideological extremity (a measure of which was constructed by folding the liberal-conservative scale at its midpoint and recoding the resulting scale to range from 0 to 1). However, recent research, exploring only the role of extremism, suggests an association between NN and support for ideological and violent extremism and violence in Indonesian, Moroccan, and Sri Lankan contexts (Jasko et al., 2020; see also Yustisia et al., 2020 for religious fundamentalism).

In summary, the interplay between political ideology and NN may display distinct patterns in Western versus Eastern Europe due to the different historical trajectories, cultural contexts, and political landscapes. On the other hand, the relationship between extremity and NN could display a more uniform pattern. Of course, understanding this interplay not only requires a deep dive into the political history and cultural dynamics of each region but also a careful consideration of the psychological mechanisms at work. As a first step in this process, we set out provide a general overview and a starting point for future, more nuanced, and causal research.

### Present Study

The overview of the complex dynamics and social consequences of political ideology and NN indicates the need to investigate further the relationships between political ideology, political extremity, and NN. Given the steady and even strengthening appeal of populism and nationalism, and radical politics (Bonikowski et al., 2019; Brubaker, 2020; Inglehart & Norris, 2017; Rooduijn & Akkerman, 2017; Seligson, 2007), this research seems especially timely. Thus, we sought to contribute to the current literature by exploring these relationships in the European context. Our effort is exploratory in nature, but based on the reviewed literature, we expected to find (at least some) evidence favoring rigidity-of-the-right and ideological extremity hypotheses on the overall sample. Furthermore, following Golec de Zavala et al.’s (2019, 65) suggestion that future research should ‘explore in more detail the relationship between collective narcissism and political conservatism in different political contexts,’ we wanted to test the differences in these relationships with respect to the political legacy by employing the distinction between Western European countries (with long histories of democratic regimes) and Eastern European countries (which transitioned from socialistic regimes in the late 1980s and early 1990s).

## Methods

### Participants

Of 51,404 participants from 69 countries and territories whose data were available in the final ICSMP COVID-19 database, we used data from 15,882 (*M*_age_ = 46.66; *SD*_age_ = 15.92; 51% females) European residents who responded to all the relevant questions, passed the attention check, and were part of samples marked as quota nationally representative with respect to age and gender.

### Measures

Political orientation was measured with a single item—participant’s self-placement on the political continuum ranging from (0) extremely left/liberal to (10) extremely right/conservative.

In line with Brandt, He, and Bender (2021; see also, for example, Van Prooijen & Kuijper, 2020 and Thorisdottir et al., 2007), we computed political (ideological) extremity as the squared scaled scores of the political orientation, with 0 indicating political moderates.

A short form of the National narcissism scale (Golec de Zavala et al., 2009) was used to measure NN (the participant’s nation represented the ingroup) as the outcome variable of this study. The scale comprised three items that measured NN (0–10 range), with higher values indicating a higher level of NN. The scale, previously validated with acceptable psychometric properties (Ardag, 2019; Sternisko et al., 2021), exhibited good internal consistency in our study (ω = .89).

We included national identification as a control for basic national, that is, ingroup attachment, and operationalized it using the item from Postmes, Haslam, and Jans (2012; *I identify as [nationality]*) and an additional item measuring identity centrality (*Being a [nationality] is an important reflection of who I am*; Cameron, 2004). Both items were measured on an 11-point scale (0 = strongly disagree, 10 = strongly agree) and were highly correlated (*r* = .69). Responses on these two items were averaged to form the scale’s total score, with higher values indicating a more robust national identification.

In addition, age, sex, and perceived personal socioeconomic status—SES (measured on a 0 to 10 ladder, with higher values denoting higher self-reported status)—were entered as control factors in the analyses. Participants from Austria, Switzerland, Denmark, Germany, Spain, France, United Kingdom, Italy, Netherlands, and Norway were grouped as participants from Western European countries. In contrast, participants from Croatia, Hungary, Latvia, Poland, Romania, Russia, Slovakia, and Ukraine were grouped as participants from Eastern European countries.

### Procedure

Data were collected within the International Collaboration on the Social and Moral Psychology of COVID-19 (Azevedo et al., 2023). The project was initiated in April 2020 via a social media call for national teams worldwide. Over 200 scholars responded to the call, including the authors of this study. The final version of the joint questionnaire was disseminated to each national team to translate into its national language and, ideally, administrate to a representative sample regarding age and sex. The data collection received an umbrella ethics approval from the University of Kent.

We performed all analyses in *R* (see Supplementary Materials), using packages lavaan (Rosseel, 2012), semTools (Jorgensen et al., 2020), psych (Revelle, 2019), ggplot2 (Wickham, 2016), and semPlot (Epskamp, 2019).

## Results

Firstly, we present the descriptive data and correlations, followed by the outputs of structural equation modeling (SEM). To account for potential differences between countries in the overall analysis and within groups, cluster-robust standard errors were calculated, providing valid results for the hypotheses at the level of individuals as multilevel models (see Hazlett & Wainstein, 2022).

As the descriptive statistics show (Table 1), participants identified with their countries on average. The responses on NN and political orientation variables were around the scales’ mid-point, suggesting that both sides of the left-right political spectrum were sufficiently represented. As can be seen, across the overall sample, NN was most strongly correlated with national identification, followed by political orientation, while its associations with sex and age were negligible and relatively low with socioeconomic status.

**Table 1:** Descriptive data and model-implied correlations between focal variables calculated on the overall sample.


		*M*	*SD*	(1)	(2)	(3)	(4)	(5)	(6)	(7)	(8)	(9)

(1)	National narcissism	–	–	–								

(2)	National narcissism – item 1	4.18	3.22	.85	-							

(3)	National narcissism – item 2	4.81	3.01	.81	.69	–						

(4)	National narcissism – item 3	4.14	3.21	.89	.76	.72	–					

(5)	Political orientation	4.80	2.28	.36	.31	.29	.33	–				

(6)	National identification	7.44	2.64	.49	.41	.39	.44	.30	–			

(7)	Sex	1.51	0.50	–.01	–.01	–.01	–.01	–.05	.05	–		

(8)	Age	46.66	15.92	.04	.04	.03	.04	<–.01	.13	–.07	–	

(9)	Socioeconomic status (SES)	5.48	1.85	.10	.08	.08	.09	–.05	.01	.07	–.03	–


*Note*. Due to the extremely large sample (*N* = 15,882), conventional significance thresholds are not marked as even meaningless correlations (e.g., *r* = .02) emerge statistically significant. Since NN is a latent variable, its *M* and *SD* are not presented.

**Figure 1:**
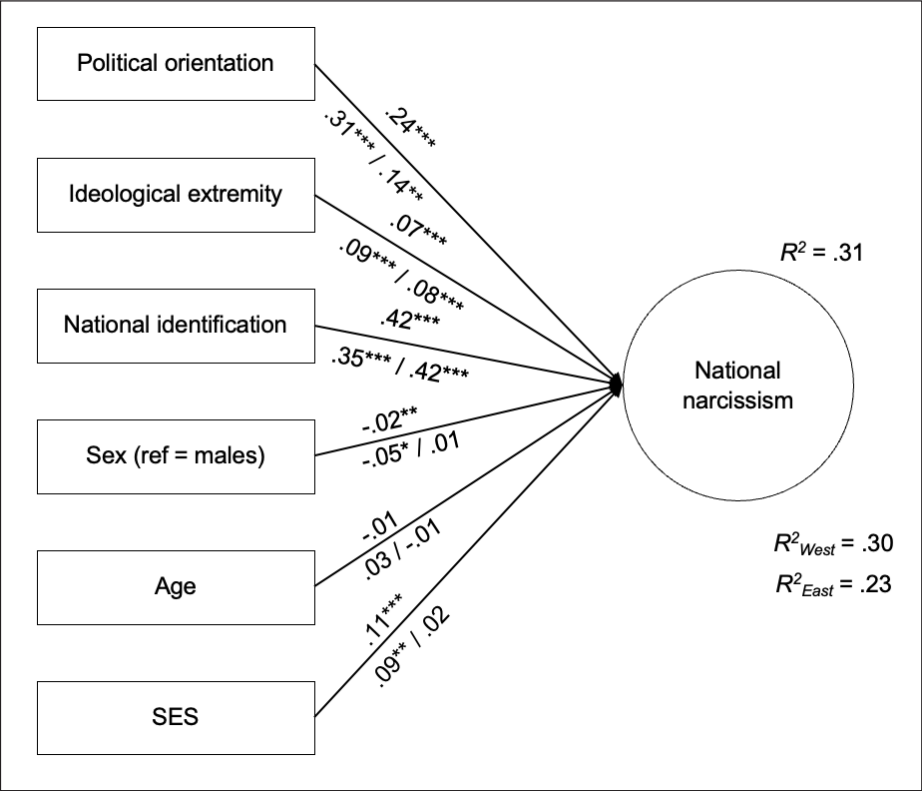
Political orientation and extremity as predictors of national narcissism. *Note*. Estimates obtained on the overall sample are presented above the paths, while estimates exhibited below the paths were obtained on the samples from Western (*n* = 9,924) and Eastern European (*n* = 5,958) countries, respectively. ***p < .001, **p < .01, *p < .05.

Next, the overall SEM model exhibited an acceptable fit (robust CFI = .997, robust RMSEA = .024, SRMR = .006). Altogether, results show that political orientation contributed substantially to explaining NN, over and above the contribution of national identification (Figure 1). The contribution of ideological extremity was weaker but significant, as was the contribution of SES, while the contribution of age and sex was negligible.

Before testing the differences in regression slopes between Eastern and Western European countries, we assessed the invariance of the entire model. According to conventional invariance criteria (Chen, 2007), strong invariance was achieved as changes in χ^2^, CFI, RMSEA, and SRMR were minimal (Table 2). However, imposing restrictions on regression slopes undermined the invariance, implying that country-groups differ regarding the relationships between predictors and the criterion.

**Table 2:** Results of invariance testing of the model predicting national narcissism across Western and Eastern European countries.


	Δχ^2^	*df*	*p*	CFI	robust RMSEA	SRMR

Configural invariance	–	–	–	.993	.030	.008

Weak invariance	4.43	2	.109	.993	.030	.009

Strong invariance	0.78	2	.679	.992	.024	.011

Invariance of regression slopes	25.53	6	<.001	.986	.031	.020


*Note*. Invariance was tested using a robust maximum likelihood estimator (MLR, Brosseau-Liard & Savalei, 2014; Brosseau-Liard, Savalei, & Li, 2012).

Analyses of regression slopes revealed a significant difference in the slopes of national identity (*z* = –2.18, *p* = .029) and political orientation across groups (*z* = 2.41, *p* = .016). In Eastern European countries, the relationship between political orientation and NN was weaker than in Western European countries and barely significant, while the relationship between national identity and NN was somewhat stronger (Figure 1). No differences in the relationship between ideological extremity and NN were found.

**Figure 2:**
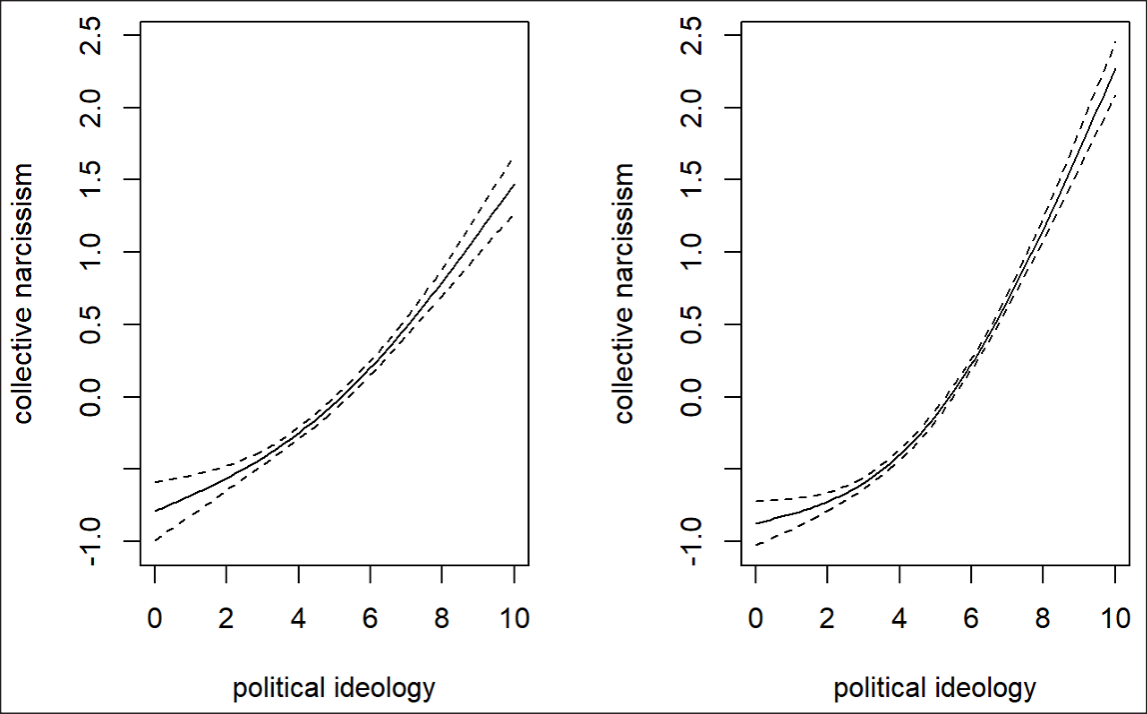
Quadratic relationship between political orientation and national narcissism (based on the constrained gam model on data comprising imputed missing values) in Eastern European countries (*n* = 5,996, left side of the figure) and Western European countries (*n* = 10,101, right side of the figure).

Three additional analyses were conducted to evaluate the robustness of these findings. Firstly, the presented analyses were re-conducted on a dataset with imputed missing values (using predictive mean matching), yielding nearly identical results. Secondly, the analyses were repeated on the dataset including imputed values with a different calculation of ideological extremity—instead of using the midpoint of the entire sample, scales were standardized with respect to the national midpoint. The outputs were again nearly identical. Thirdly, we applied generalized additive model (gam) analyses on the dataset comprising imputed values to additionally evaluate the nature of the non-linear relationship between political ideology and NN. National narcissism factor scores were extracted from models and used as the criteria, while all the predictors were simultaneously included in the regression. Both models included the interaction between the smooth term of political orientation and region of Europe (East vs. West). In the first model, we did not impose any constraints on smoothing, while in the second model we limited the smoothing to the use of only two functions (*k* = 2). The outcomes of the first model suggested very complex functions: smooth terms calculated for both Eastern and Western European countries were significant with the effective degrees of freedom being around seven (edf_East_ = 7.50, edf_West_ = 7.04). Such findings suggest that a very complex curve is required to describe the relationship between political ideology and NN. The second model was used to evaluate whether the relationship between NN and political orientation could be well explained even with a simpler curve—a quadratic curve. The outcomes of analyses provided arguments in favor of this notion: the effective degrees of freedom were significant and close to two, implying an almost perfect quadratic relationship (edf_East_ = 1.94, edf_West_ = 1.99). Furthermore, *p*-values of the tests of residuals were insignificant, implying that the quadratic relationship has not failed to capture any strong trends. Considering the minimal differences in the explained deviance of the two models (*R*^2^_adjusted_ = .28 for unconstrained model 1 and *R*^2^_adjusted_ = .27 for model 2 with a constrained number of functions), it seems that the quadratic relationship represents a useful (although slightly imperfect) tool for depicting the relationship between political ideology and NN. These relationships are visualized in Figure 2, while a detailed presentation of analytical outcomes can be found in the Supplementary materials.

## Discussion

In the present study, we investigated how adherence to left and right ideology is associated with NN in different socio-political contexts. Namely, by analyzing quadratic and linear relationships between political orientation (as a predictor) and NN (as a criterion), we were able to evaluate whether NN was more characteristic of the political right or left, thus testing the propositions derived from both the *ideological extremity* and *rigidity-of-the-right* approaches.

Overall, regarding individuals’ inclination to NN, evidence provides more support for the notion of the psychological rigidity of the right. Specifically, the outcomes revealed that both political orientation and ideological extremity significantly explained NN (although weaker than national identification, which served as a controlling factor). However, the linear relationships (political orientation) seem much stronger than the non-linear ones (ideological extremity). Such results imply that incorporating the curve only slightly improves the prediction over the linear models, and, as the presented graphs suggest, their combination is not a U-shaped curve, but rather a weak curve tilted to one side. Thus, the outcomes of regression analyses revealed that right-leaning individuals, on average, scored the highest on NN, implying that NN is a characteristic of the political right. This is consistent with previous studies on NN (Cislak et al., 2020; Golec de Zavala & Federico, 2018) and the lines of research suggesting that the political left and right differ in their underlying psychological underpinnings that drive political ideology (e.g., Graham, Haidt, & Nosek, 2009; Jost, 2017; Napier & Jost, 2008).

Following Golec de Zavala et al.’s (2019, 65) suggestion of exploring the relationship between NN and political conservatism across different political contexts, we explored it as a possible moderating factor in our research. Specifically, the analyses indicate that the relationships between political orientation and extremity with NN vary somewhat across the two European socio-cultural backgrounds. The regression outcomes revealed that the relationship between political orientation and NN was stronger in Western European countries (compared to Eastern European countries), indicating that NN is a characteristic of the right-leaning nationals in those countries. This is more consistent with research suggesting that rigidity of the right is more common, especially in Western European countries with a history of liberalism and capitalism (e.g., Jost et al., 2003a; Thorisdottir et al., 2007). The weaker relationship between political orientation and NN established in Eastern European countries suggests that in those countries, the political left and right do not differ as much in terms of NN. Countries grouped as Eastern European for the purposes of this study have experienced socialism and communism, with many of them being under similar political regimes until the last decade of the twentieth century. The egalitarian principles promoted during this period may have provided social and economic security (Flanagan et al., 2003; Mieriņa, 2018). In those countries, as Thorisdottir et al. (2007) argue, it seems that a preference for inequality is more driven by an acceptance of risk than by the need for security. Thus, it might be the case that in societies without past or current ties to communism, the left-leaning ideological extreme is oriented toward human liberties and equality. At the same time, the experience of socialist regimes could have resulted in the association of the political left with more authoritarian values (Costello et al., 2022), which was still evident during the time of data collection.

Several limitations should be considered when interpreting the results. Firstly, as with all cross-sectional, correlational research, a limitation of our approach is that no causal inferences can be inferred. Furthermore, the study was conducted during the COVID-19 pandemic, representing a heightened threat context, and threats tend to strengthen the relationship between ingroup identification and ingroup bias, as well as social identification with the ingroup (Voci, 2006). Furthermore, national identity (Kunovich, 2009) and political orientation (Bauer et al., 2017) are multifaceted phenomena that can be a source of non-systematic variability in large, transnational studies. This is further complicated by findings that the consistency of positioning on the political right and left is questionable even within a single country (Bauer et al., 2017). Thus, in future studies, we recommend using more refined measures that allow more detailed testing of the linear, non-linear, and interactive relationships between political orientation, ideological extremity, and NN. Finally, future studies could also benefit from including variables that reflect political sophistication or knowledge, which were unavailable in the dataset used for our analyses. In that vein, we controlled for SES. Further investigation of ‘nominal centrists’ (respondents who select the midpoint on a left-right ideological scale), often characterized by their limited political knowledge and engagement (Rodon, 2015) which in fact may be indicative of a subtler form of non-response or disengagement from the political spectrum (Scholz & Zuell, 2016), is required.

## Conclusion

We sought to contribute to understanding the relationship between political ideology, political extremity, and national narcissism. Namely, we wanted to provide a general overview and a starting point for future, more nuanced, and causal research. In the study, we tested two hypotheses related to NN: rigidity-of-the-right and ideological extremity. Consistent with Jost et al.’s (2003a) proposal, the results strongly support the rigidity-of-the-right hypothesis. Furthermore, although the evidence shows that socio-political context should be considered to fully understand the relationship between political ideology, political extremity, and national narcissism, the rigidity-of-the-right hypothesis was confirmed both in Eastern and Western European countries. Such results imply that regardless of the cultural (and historical) background, right-leaning European citizens tend to exhibit higher collective narcissism than centrists and left-leaning citizens. Our study highlights the role of right-leaning political orientation in understanding national narcissism, offering a foundation for future investigations into the psychological mechanisms underlying this relationship and its potential impact on societal dynamics and international relations.

## Data Accessibility Statement

Data descriptor article by Azevedo et al. (2023) presents the dataset, which includes data from 51,404 individuals across 69 countries, associated with the ICSMP COVID-19 project. All materials related to the project are available in the project’s repository (consisting of five folders) hosted by the Open Science Framework (OSF, https://doi.org/10.17605/osf.io/tfsza).

Of the total of 51,404 participants, we used the data from 15,882 European residents who responded to all the relevant questions, passed the attention check, and were part of samples marked as quota nationally representative with respect to age and gender.

## Additional File

The additional file for this article can be found as follows:

10.5334/irsp.844.s1 Supplementary File 1. Supplemental Material—Data analyses.
