# Supplementary material for: Making Nations Great Again: National Narcissism and the Left, the Right, and the Extreme in the European Context
Source: Int Rev Soc Psychol. 2024 Feb 12;37:2. doi: 10.5334/irsp.844 (PMC12372775; doi:10.5334/irsp.844)

Collective narcissism and national identification

Deadpool

19 9 2023

Preparations

```
sapply(c("dplyr", "readr", "haven", "psych", "lavaan", "semTools", "ggplot2", "seminr", "mice"), librar

spinburst <- function(model, decimals = 4, sig_level = .05){
  slopes <- parameterestimates(model, standardized = T) #extracts parameter estimates
  slps <- as.data.frame(slopes[slopes$op == "~", ]) #keeps only regressions
  slps$combos <- paste(slps$lhs, "predicted by", slps$rhs)
  spinned <- split.data.frame(slps, slps$combos)
  nam <- names(spinned)
  for(l in seq_along(spinned)){
    df <- spinned[[l]]
    xyz <- list()
    for(i in 1:nrow(df)){
      yzx <- list()
      for(j in 1:nrow(df)){
        if(i != j){
          x <- df[i, "est"]
          xs <- df[i, "se"]
          y <- df[j, "est"]
          ys <- df[j, "se"]
          z <- (x-y)/sqrt(xs^2+ys^2)
          yzx[j] <- z
        } else {
          yzx[j] <- NA
        }
      }
      xyz[[i]] <- yzx
    } #lvl 2 for loop to make all the comparisons
    f <- round(as.numeric(unlist(xyz)), decimals) #rounded z-values of comparisons
    thresh <- abs(qnorm(sig_level/(length(unique(f))-1))) #Bonferroni-adjusted significance threshold
    ff <- ifelse(f < thresh & f > -thresh, "no", f) #marking non-significant differences
    yyy <- matrix(ff, byrow = length(unique(df$group)), ncol = length(unique(df$group))) #forming a mat
    zzz <- as.data.frame(yyy) #forming a data frame
    rownames(zzz) <- lavInspect(model, "group.label") #renaming rows
    colnames(zzz) <- lavInspect(model, "group.label") #renaming columns
    message(c("Homogeneous subsets according to significance testing:", nam[l]))
    fw <- rep(lavInspect(model, "group.label"), each = length(lavInspect(model, "group.label")))
    fwf <- matrix(fw, byrow = length(unique(df$group)), ncol = length(unique(df$group)))
    fwf[zzz != "no"] <- NA
    fwdf <- t(as.data.frame(fwf))
```

```
    ffwdf <- fwdf[duplicated(fwdf) == F, ]
    ffwdf[is.na(ffwdf)] <- "  "
    print(ffwdf)
    message("Table with z-values of testing the differences between slopes (Bonferroni adjusted):", nam
    zzz[lower.tri(zzz)] <- ""
    print(zzz)
    oOo <- df[, c("group", "est", "se", "pvalue", "std.all")]
    oOo$group <- lavInspect(model, "group.label")
    message("Original coefficients:", nam[1])
    OoO <- cbind(oOo[,1], round(oOo[, 2:5], decimals))
    names(OoO)[1] <- "group"
    names(OoO)[5] <- "beta"
    print(OoO)
  }
}
```

Data manipulations

```
c19 <- read_sav("ICSMP_cleaned_data_with_dates.sav", encoding = "latin1")
c19$comm <- ifelse(c19$ISO3 %in% c("BGR", "HRV", "HUN", "LVA", "MKD", "POL", "ROU", "RUS", "SRB", "SVK"
c19 <- subset(c19, comm %in% c("East Europe (N = 5958)", "West Europe (N = 9924)"))
cor(c19$nidentity1, c19$nidentity2, use = "pairwise.complete.obs")
```

```
## [1] 0.6868841
```

```
c19$natid <- rowMeans(c19[, c("nidentity1", "nidentity2")])
c19$cnarc1 <- as.numeric(c19$cnarc1)
c19$cnarc2 <- as.numeric(c19$cnarc2)
c19$cnarc3 <- as.numeric(c19$cnarc3)
c20 <- c19[,c("att_check_nobots", "ISO3", "revision_coding", "comm", "natid", "cnarc1", "cnarc2", "cnar
c20 <- subset(c20, revision_coding == 1 & att_check_nobots == 1)
dim(c20)
```

```
## [1] 16144    12
```

```
c20$polid2 <- scale(c20$political_ideology)*scale(c20$political_ideology)
write.csv(c20, "forimputations.csv")
c20 <- c20[complete.cases(c20), ]
describe(c20)
```

```
##                    vars     n  mean    sd median trimmed   mad   min    max
## att_check_nobots      1 15882  1.00  0.00   1.00    1.00  0.00  1.00   1.00
## ISO3*                 2 15882  8.61  5.09   9.00    8.57  7.41  1.00  17.00
## revision_coding       3 15882  1.00  0.00   1.00    1.00  0.00  1.00   1.00
## comm*                 4 15882  1.62  0.48   2.00    1.66  0.00  1.00   2.00
## natid                 5 15882  7.44  2.64   8.00    7.84  2.97  0.00  10.00
## cnarc1                6 15882  4.18  3.22   5.00    4.00  4.45  0.00  10.00
## cnarc2                7 15882  4.81  3.01   5.00    4.78  2.97  0.00  10.00
## cnarc3                8 15882  4.14  3.21   5.00    3.96  4.45  0.00  10.00
## political_ideology    9 15882  4.80  2.28   5.00    4.78  2.97  0.00  10.00
```

```
## sex1                  10 15882  1.51  0.50   2.00    1.51  0.00  1.00   3.00
## age                   11 15882 46.66 15.92  47.00   46.58 19.27 18.00 100.00
## ladder                12 15882  5.48  1.85   6.00    5.43  1.48  1.00  11.00
## polid2                13 15882  1.00  1.34   0.62    0.71  0.90  0.01   5.19
##                     range  skew kurtosis   se
## att_check_nobots     0.00   NaN      NaN 0.00
## ISO3*               16.00  0.00    -1.37 0.04
## revision_coding      0.00   NaN      NaN 0.00
## comm*                1.00 -0.52    -1.73 0.00
## natid               10.00 -1.01     0.27 0.02
## cnarc1              10.00  0.17    -1.04 0.03
## cnarc2              10.00 -0.10    -0.83 0.02
## cnarc3              10.00  0.18    -1.05 0.03
## political_ideology 10.00  0.06    -0.20 0.02
## sex1                 2.00  0.02    -1.90 0.00
## age                 82.00  0.06    -0.97 0.13
## ladder              10.00  0.28     0.31 0.01
## polid2               5.19  1.68     2.09 0.01
```

```
c20$PoliticId <- c20$political_ideology

table(c20$comm)
```

```
##
## East Europe (N = 5958) West Europe (N = 9924)
##                   5958                   9924
```

```
table(c20$comm, c20$ISO3)
```

```
##
##                           AUT  CHE  DEU  DNK  ESP  FRA  GBR  HRV  HUN  ITA  LVA
##   East Europe (N = 5958)    0    0    0    0    0    0    0  501  494    0  996
##   West Europe (N = 9924) 1328 1053 1548  553 1074 1091  544    0    0  949    0
##
##                           NLD  NOR  POL  RUS  SVK  UKR
##   East Europe (N = 5958)    0    0 1800  507 1083  577
##   West Europe (N = 9924) 1271  513    0    0    0    0
```

Main analysis

```
model <- 'cnar =~ cnarc1 + cnarc2 + cnarc3
          cnar ~ PoliticId + polid2 + natid + sex1 + age + ladder'

modmod <- sem(model, estimator = "MLR", data = c20)
psych::omega(c20[,c("cnarc1", "cnarc2", "cnarc3")])
```

```
## Loading required namespace: GPArotation
```

```
## Warning in cov2cor(t(w) %*% r %*% w): diag(.) had 0 or NA entries; non-finite
## result is doubtful
```

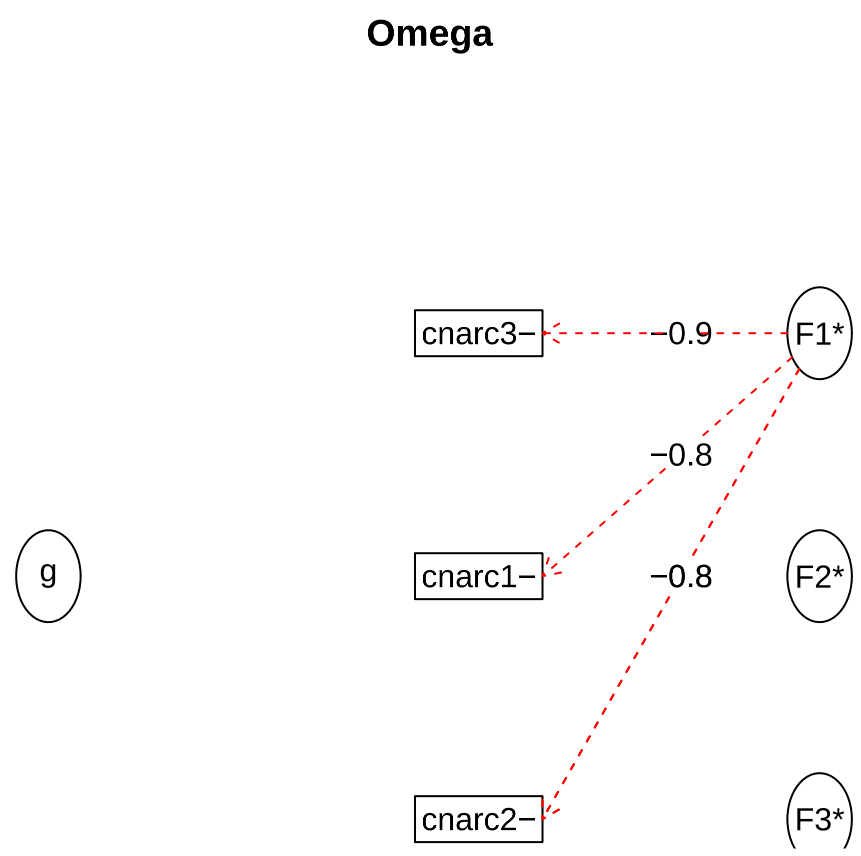


```
## Omega
## Call: omegah(m = m, nfactors = nfactors, fm = fm, key = key, flip = flip,
##     digits = digits, title = title, sl = sl, labels = labels,
##     plot = plot, n.obs = n.obs, rotate = rotate, Phi = Phi, option = option,
##     covar = covar)
## Alpha:                 0.89
## G.6:                   0.84
## Omega Hierarchical:    0.02
## Omega H asymptotic:    0.03
## Omega Total            0.89
##
## Schmid Leiman Factor loadings greater than  0.2
##             g   F1*   F2*   F3*   h2   u2   p2
## cnarc1-       -0.84             0.73 0.27 0.02
## cnarc2-       -0.79             0.65 0.35 0.03
## cnarc3-       -0.88             0.80 0.20 0.03
##
## With Sums of squares  of:
##    g  F1*  F2*  F3*
## 0.06 2.12 0.01 0.00
##
## general/max  0.03   max/min =   Inf
## mean percent general =  0.03    with sd =  0.01 and cv of  0.22
## Explained Common Variance of the general factor =  0.03
##
## The degrees of freedom are -3  and the fit is  0
```

```
## The number of observations was  15882  with Chi Square =  0  with prob <  NA
## The root mean square of the residuals is  0
## The df corrected root mean square of the residuals is  NA
##
## Compare this with the adequacy of just a general factor and no group factors
## The degrees of freedom for just the general factor are 0  and the fit is  1.62
## The number of observations was  15882  with Chi Square =  25687.5  with prob <  NA
## The root mean square of the residuals is  0.7
## The df corrected root mean square of the residuals is  NA
##
## Measures of factor score adequacy
##                                                  g  F1*   F2* F3*
## Correlation of scores with factors             0.15 0.93  0.17   0
## Multiple R square of scores with factors       0.02 0.87  0.03   0
## Minimum correlation of factor score estimates -0.95 0.75 -0.94  -1
##
##  Total, General and Subset omega for each subset
##                                                 g  F1* F2* F3*
## Omega total for total scores and subscales    0.89 0.89  NA  NA
## Omega general for total scores and subscales  0.02 0.02  NA  NA
## Omega group for total scores and subscales    0.87 0.87  NA  NA
```

```
summary(modmod, standardized = T, rsq = T, fit.measures = T)
```

```
## lavaan 0.6.15 ended normally after 36 iterations
##
##   Estimator                                         ML
##   Optimization method                           NLMINB
##   Number of model parameters                        12
##
##   Number of observations                         15882
##
## Model Test User Model:
##                                               Standard      Scaled
##   Test Statistic                               118.912     108.695
##   Degrees of freedom                                12          12
##   P-value (Chi-square)                           0.000       0.000
##   Scaling correction factor                                  1.094
##     Yuan-Bentler correction (Mplus variant)
##
## Model Test Baseline Model:
##
##   Test statistic                             32171.021   25321.900
##   Degrees of freedom                                21          21
##   P-value                                        0.000       0.000
##   Scaling correction factor                                  1.270
##
## User Model versus Baseline Model:
##
##   Comparative Fit Index (CFI)                    0.997       0.996
##   Tucker-Lewis Index (TLI)                       0.994       0.993
##
##   Robust Comparative Fit Index (CFI)                         0.997
##   Robust Tucker-Lewis Index (TLI)                            0.994
```

```
##
## Loglikelihood and Information Criteria:
##
##   Loglikelihood user model (H0)           -106146.769 -106146.769
##   Scaling correction factor                                 1.258
##       for the MLR correction
##   Loglikelihood unrestricted model (H1)             NA          NA
##   Scaling correction factor                                 1.176
##       for the MLR correction
##
##   Akaike (AIC)                             212317.538  212317.538
##   Bayesian (BIC)                           212409.613  212409.613
##   Sample-size adjusted Bayesian (SABIC)    212371.478  212371.478
##
## Root Mean Square Error of Approximation:
##
##   RMSEA                                         0.024       0.023
##   90 Percent confidence interval - lower        0.020       0.019
##   90 Percent confidence interval - upper        0.028       0.026
##   P-value H_0: RMSEA <= 0.050                   1.000       1.000
##   P-value H_0: RMSEA >= 0.080                   0.000       0.000
##
##   Robust RMSEA                                              0.024
##   90 Percent confidence interval - lower                    0.020
##   90 Percent confidence interval - upper                    0.028
##   P-value H_0: Robust RMSEA <= 0.050                        1.000
##   P-value H_0: Robust RMSEA >= 0.080                        0.000
##
## Standardized Root Mean Square Residual:
##
##   SRMR                                          0.006       0.006
##
## Parameter Estimates:
##
##   Standard errors                             Sandwich
##   Information bread                           Observed
##   Observed information based on                Hessian
##
## Latent Variables:
##                    Estimate  Std.Err  z-value  P(>|z|)   Std.lv  Std.all
##   cnar =~
##     cnarc1            1.000                               2.733    0.850
##     cnarc2            0.888    0.008  111.912    0.000    2.427    0.806
##     cnarc3            1.051    0.007  143.672    0.000    2.873    0.895
##
## Regressions:
##                    Estimate  Std.Err  z-value  P(>|z|)   Std.lv  Std.all
##   cnar ~
##     PoliticId         0.286    0.010   28.757    0.000    0.105    0.239
##     polid2            0.151    0.016    9.583    0.000    0.055    0.074
##     natid             0.436    0.008   51.408    0.000    0.160    0.420
##     sex1             -0.124    0.039   -3.152    0.002   -0.045   -0.023
##     age              -0.002    0.001   -1.470    0.141   -0.001   -0.011
##     ladder            0.156    0.011   14.042    0.000    0.057    0.105
```

```
##
## Variances:
##                    Estimate  Std.Err  z-value  P(>|z|)   Std.lv  Std.all
##    .cnarc1            2.869    0.068   42.100    0.000    2.869    0.278
##    .cnarc2            3.165    0.067   47.291    0.000    3.165    0.350
##    .cnarc3            2.051    0.064   32.229    0.000    2.051    0.199
##    .cnar              5.176    0.081   63.764    0.000    0.693    0.693
##
## R-Square:
##                    Estimate
##     cnarc1            0.722
##     cnarc2            0.650
##     cnarc3            0.801
##     cnar              0.307
```

```
as.data.frame(lavInspect(modmod, "cor.all"))
```

```
##                cnarc1      cnarc2      cnarc3    PoliticId       polid2
## cnarc1     1.00000000  0.68549750  0.76068630  0.309982264  0.058012733
## cnarc2     0.68549750  1.00000000  0.72177377  0.294125277  0.055045120
## cnarc3     0.76068630  0.72177377  1.00000000  0.326386411  0.061082745
## PoliticId  0.30998226  0.29412528  0.32638641  1.000000000  0.044811581
## polid2     0.05801273  0.05504512  0.06108274  0.044811581  1.000000000
## natid      0.41466222  0.39345038  0.43660599  0.301237303 -0.039650837
## sex1      -0.01057241 -0.01003158 -0.01113190 -0.052144398 -0.058498873
## age        0.03569439  0.03386847  0.03758333 -0.004951683  0.002824455
## ladder     0.08116711  0.07701505  0.08546245 -0.047712290 -0.011980571
## cnar       0.84997311  0.80649316  0.89495337  0.364696554  0.068252433
##                  natid        sex1          age       ladder         cnar
## cnarc1     0.414662220 -0.01057241  0.035694394  0.081167112  0.84997311
## cnarc2     0.393450382 -0.01003158  0.033868465  0.077015049  0.80649316
## cnarc3     0.436605991 -0.01113190  0.037583328  0.085462445  0.89495337
## PoliticId  0.301237303 -0.05214440 -0.004951683 -0.047712290  0.36469655
## polid2    -0.039650837 -0.05849887  0.002824455 -0.011980571  0.06825243
## natid      1.000000000  0.04537232  0.130212252  0.009178676  0.48785334
## sex1       0.045372318  1.00000000 -0.073796222  0.069105928 -0.01243852
## age        0.130212252 -0.07379622  1.000000000 -0.027187177  0.04199473
## ladder     0.009178676  0.06910593 -0.027187177  1.000000000  0.09549374
## cnar       0.487853339 -0.01243852  0.041994734  0.095493741  1.00000000
```

```
modmodc <- sem(model, estimator = "MLR", data = c20, group = "comm", cluster = "ISO3")
```

```
## Warning in lav_model_vcov(lavmodel = lavmodel, lavsamplestats = lavsamplestats, : lavaan WARNING:
##     The variance-covariance matrix of the estimated parameters (vcov)
##     does not appear to be positive definite! The smallest eigenvalue
##     (= -1.099638e-14) is smaller than zero. This may be a symptom that
##     the model is not identified.
```

```
modmodw <- sem(model, estimator = "MLR", data = c20, group = "comm", group.equal = "loadings", cluster =
```

```
## Warning in lav_model_vcov(lavmodel = lavmodel, lavsamplestats = lavsamplestats, : lavaan WARNING:
##     The variance-covariance matrix of the estimated parameters (vcov)
```

```
##     does not appear to be positive definite! The smallest eigenvalue
##     (= -7.505242e-16) is smaller than zero. This may be a symptom that
##     the model is not identified.
```

```r
modmods <- sem(model, estimator = "MLR", data = c20, group = "comm", group.equal = c("loadings", "inter
```

```
## Warning in lav_model_vcov(lavmodel = lavmodel, lavsamplestats = lavsamplestats, : lavaan WARNING:
##     The variance-covariance matrix of the estimated parameters (vcov)
##     does not appear to be positive definite! The smallest eigenvalue
##     (= -2.206918e-16) is smaller than zero. This may be a symptom that
##     the model is not identified.
```

```r
modmodr <- sem(model, estimator = "MLR", data = c20, group = "comm", group.equal = c("loadings", "inter
```

```
## Warning in lav_model_vcov(lavmodel = lavmodel, lavsamplestats = lavsamplestats, : lavaan WARNING:
##     The variance-covariance matrix of the estimated parameters (vcov)
##     does not appear to be positive definite! The smallest eigenvalue
##     (= 3.302437e-16) is close to zero. This may be a symptom that the
##     model is not identified.
```

```r
summary(compareFit(modmodc, modmodw, modmods, modmodr, nested = T), fit.measures = c("cfi", "cfi.robust
```

```
## ################### Nested Model Comparison ########################
##
## Scaled Chi-Squared Difference Test (method = "satorra.bentler.2001")
##
## lavaan NOTE:
##     The "Chisq" column contains standard test statistics, not the
##     robust test that should be reported per model. A robust difference
##     test is a function of two standard (not robust) statistics.
##
##         Df    AIC    BIC  Chisq Chisq diff Df diff Pr(>Chisq)
## modmodc 24 211245 211475 220.21
## modmodw 26 211261 211476 239.82     4.4255       2   0.109401
## modmods 28 211289 211488 271.75     0.7752       2   0.678693
## modmodr 34 211446 211600 441.07    25.5327       6   0.000272 ***
## ---
## Signif. codes:  0 '***' 0.001 '**' 0.01 '*' 0.05 '.' 0.1 ' ' 1
##
## ####################### Model Fit Indices ###########################
##           cfi cfi.robust rmsea.robust  srmr
## modmodc .993†        NA         .030  .008†
## modmodw .993         NA         .030  .009
## modmods .992         NA         .024† .011
## modmodr .986         NA         .031  .020
##
## ################## Differences in Fit Indices #######################
##                       cfi cfi.robust rmsea.robust  srmr
## modmodw - modmodc -0.001         NA        0.000 0.001
## modmods - modmodw -0.001         NA       -0.005 0.001
## modmodr - modmods -0.006         NA        0.007 0.010
```

```r
summary(modmods, standardized = T, rsq = T)
```

```
## lavaan 0.6.15 ended normally after 98 iterations
##
##   Estimator                                         ML
##   Optimization method                           NLMINB
##   Number of model parameters                        31
##   Number of equality constraints                     5
##
##   Number of observations per group:
##     West Europe (N = 9924)                        9924
##     East Europe (N = 5958)                        5958
##   Number of clusters [ISO3]:
##     West Europe (N = 9924)                          10
##     East Europe (N = 5958)                           7
##
## Model Test User Model:
##                                               Standard      Scaled
##   Test Statistic                               271.753      54.340
##   Degrees of freedom                                28          28
##   P-value (Chi-square)                           0.000       0.002
##   Scaling correction factor                                  5.001
##     Yuan-Bentler correction (Mplus variant)
##   Test statistic for each group:
##     West Europe (N = 9924)                     152.137      30.421
##     East Europe (N = 5958)                     119.616      23.918
##
## Parameter Estimates:
##
##   Standard errors                           Robust.cluster
##   Information                                     Observed
##   Observed information based on                    Hessian
##
##
## Group 1 [West Europe (N = 9924)]:
##
## Latent Variables:
##                    Estimate  Std.Err  z-value  P(>|z|)   Std.lv  Std.all
##   cnar =~
##     cnarc1            1.000                               2.594    0.843
##     cnarc2  (.p2.)    0.886    0.044   20.274    0.000    2.298    0.787
##     cnarc3  (.p3.)    1.051    0.017   62.145    0.000    2.726    0.894
##
## Regressions:
##                    Estimate  Std.Err  z-value  P(>|z|)   Std.lv  Std.all
##   cnar ~
##     PoliticId         0.344    0.039    8.897    0.000    0.132    0.306
##     polid2            0.192    0.054    3.545    0.000    0.074    0.094
##     natid             0.335    0.047    7.070    0.000    0.129    0.351
##     sex1             -0.230    0.110   -2.086    0.037   -0.089   -0.045
##     age               0.005    0.005    0.958    0.338    0.002    0.028
##     ladder            0.128    0.049    2.605    0.009    0.049    0.091
##
```

```
## Intercepts:
##                    Estimate  Std.Err  z-value  P(>|z|)   Std.lv  Std.all
##    .cnarc1  (.35.)   -1.182    0.562   -2.102    0.036   -1.182   -0.384
##    .cnarc2  (.36.)    0.059    0.535    0.111    0.912    0.059    0.020
##    .cnarc3  (.37.)   -1.490    0.620   -2.403    0.016   -1.490   -0.489
##    .cnar              0.000                               0.000    0.000
##
## Variances:
##                    Estimate  Std.Err  z-value  P(>|z|)   Std.lv  Std.all
##    .cnarc1            2.735    0.165   16.528    0.000    2.735    0.289
##    .cnarc2            3.250    0.296   10.978    0.000    3.250    0.381
##    .cnarc3            1.876    0.135   13.924    0.000    1.876    0.202
##    .cnar              4.680    0.521    8.988    0.000    0.696    0.696
##
## R-Square:
##                    Estimate
##     cnarc1            0.711
##     cnarc2            0.619
##     cnarc3            0.798
##     cnar              0.304
##
##
## Group 2 [East Europe (N = 5958)]:
##
## Latent Variables:
##                    Estimate  Std.Err  z-value  P(>|z|)   Std.lv  Std.all
##   cnar =~
##     cnarc1            1.000                               2.552    0.825
##     cnarc2  (.p2.)    0.886    0.044   20.274    0.000    2.261    0.790
##     cnarc3  (.p3.)    1.051    0.017   62.145    0.000    2.682    0.868
##
## Regressions:
##                    Estimate  Std.Err  z-value  P(>|z|)   Std.lv  Std.all
##   cnar ~
##     PoliticId         0.167    0.062    2.688    0.007    0.065    0.144
##     polid2            0.138    0.037    3.721    0.000    0.054    0.078
##     natid             0.468    0.038   12.163    0.000    0.183    0.418
##     sex1              0.043    0.053    0.819    0.413    0.017    0.009
##     age              -0.002    0.004   -0.572    0.568   -0.001   -0.014
##     ladder            0.024    0.055    0.444    0.657    0.010    0.017
##
## Intercepts:
##                    Estimate  Std.Err  z-value  P(>|z|)   Std.lv  Std.all
##    .cnarc1  (.35.)   -1.182    0.562   -2.102    0.036   -1.182   -0.382
##    .cnarc2  (.36.)    0.059    0.535    0.111    0.912    0.059    0.021
##    .cnarc3  (.37.)   -1.490    0.620   -2.403    0.016   -1.490   -0.483
##    .cnar              1.588    0.877    1.810    0.070    0.622    0.622
##
## Variances:
##                    Estimate  Std.Err  z-value  P(>|z|)   Std.lv  Std.all
##    .cnarc1            3.047    0.157   19.436    0.000    3.047    0.319
##    .cnarc2            3.069    0.224   13.677    0.000    3.069    0.375
##    .cnarc3            2.346    0.150   15.606    0.000    2.346    0.246
##    .cnar              5.041    0.524    9.618    0.000    0.774    0.774
```

```
##
## R-Square:
##                    Estimate
##     cnarc1            0.681
##     cnarc2            0.625
##     cnarc3            0.754
##     cnar              0.226
```

```
model <- 'cnar =~ cnarc1 + cnarc2 + cnarc3'
modmod <- sem(model, estimator = "MLR", data = c20)

df <- as.data.frame(estimate_lavaan_ten_berge(modmod)$scores)
df2 <- cbind(c20, df)

#overall
ggplot(df2, aes(x = PoliticId, y = cnar)) + geom_jitter(alpha = .2, col = "gray50") + stat_smooth(metho
```

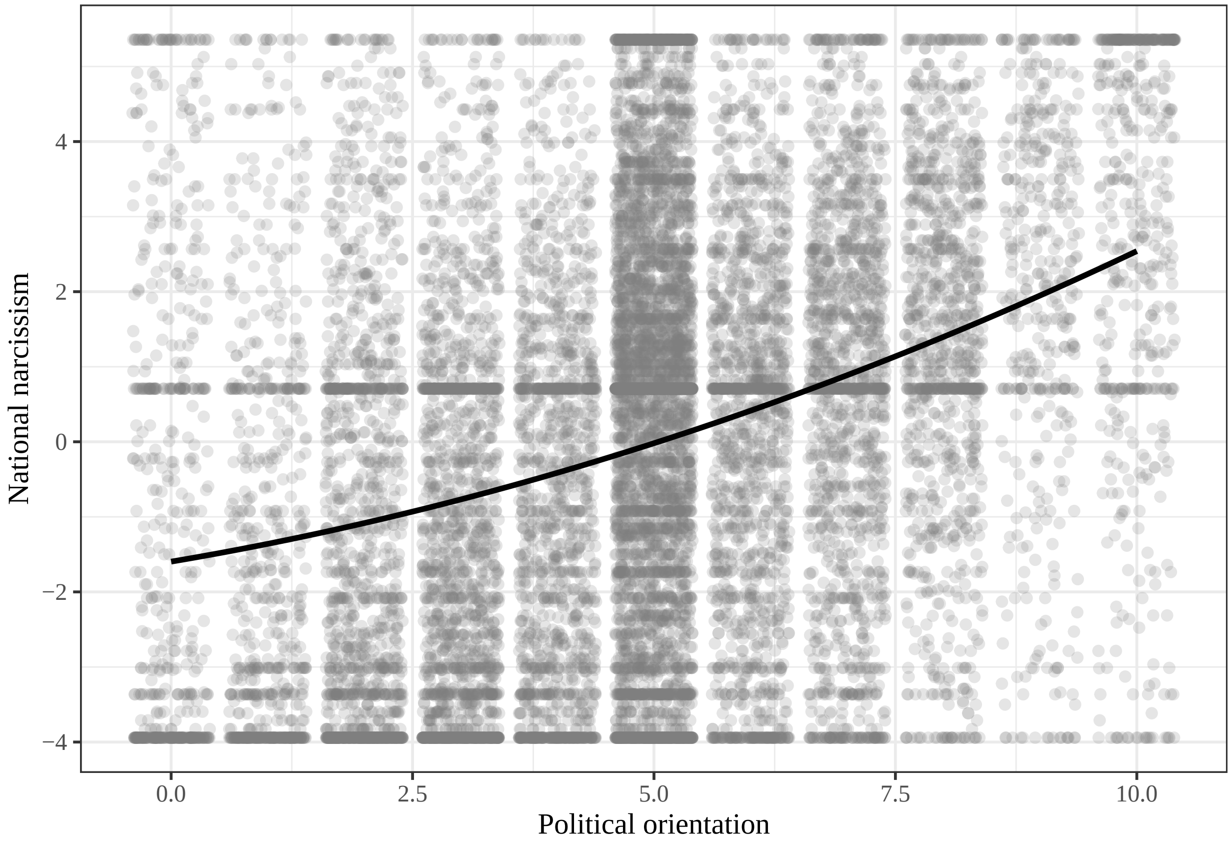


```
ggplot(df2, aes(x = PoliticId, y = cnar)) + geom_jitter(alpha = .2, col = "gray50") + stat_smooth(metho
```

```
## `geom_smooth()` using formula = 'y ~ s(x, bs = "cs")'
```

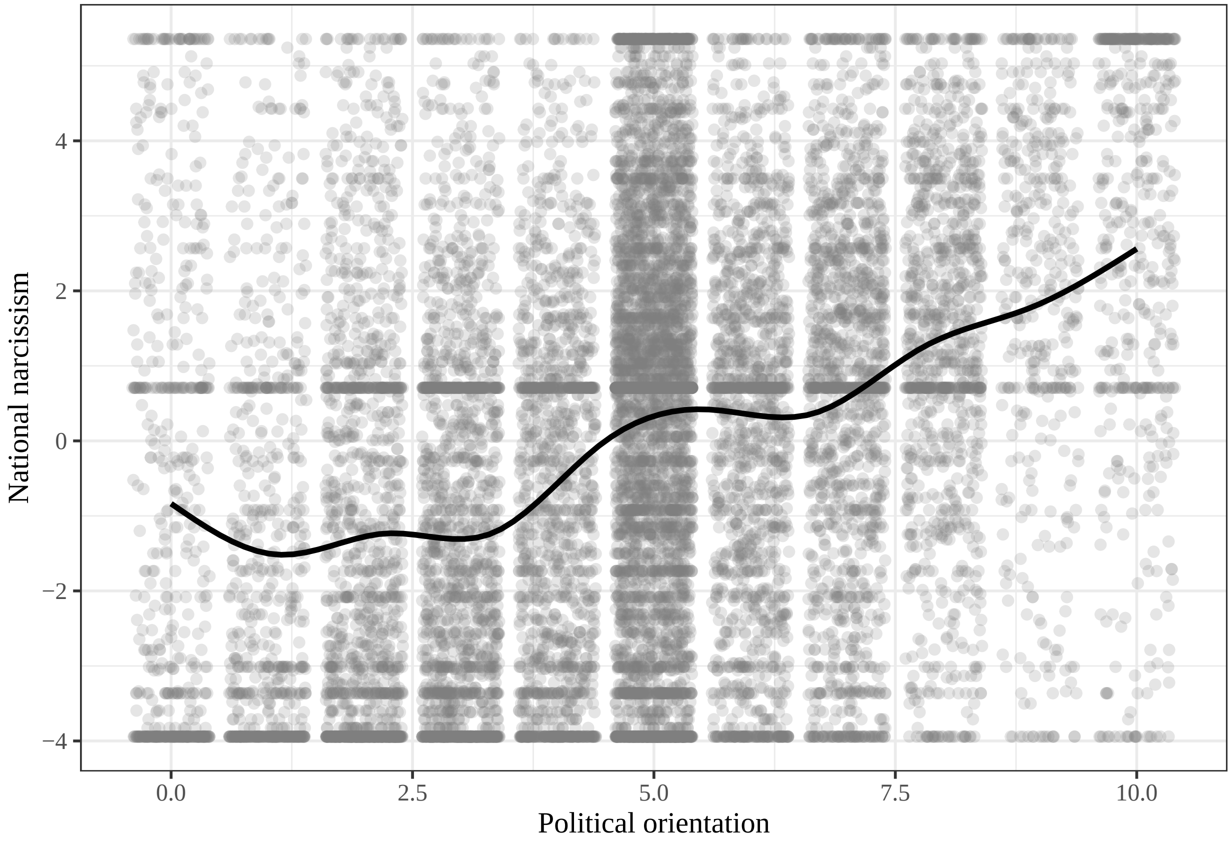


```r
#linear
ggplot(df2, aes(x = PoliticId, y = cnar, color = comm)) + geom_jitter(alpha = .3) + geom_smooth(method =
```

```
## `geom_smooth()` using formula = 'y ~ x'
```

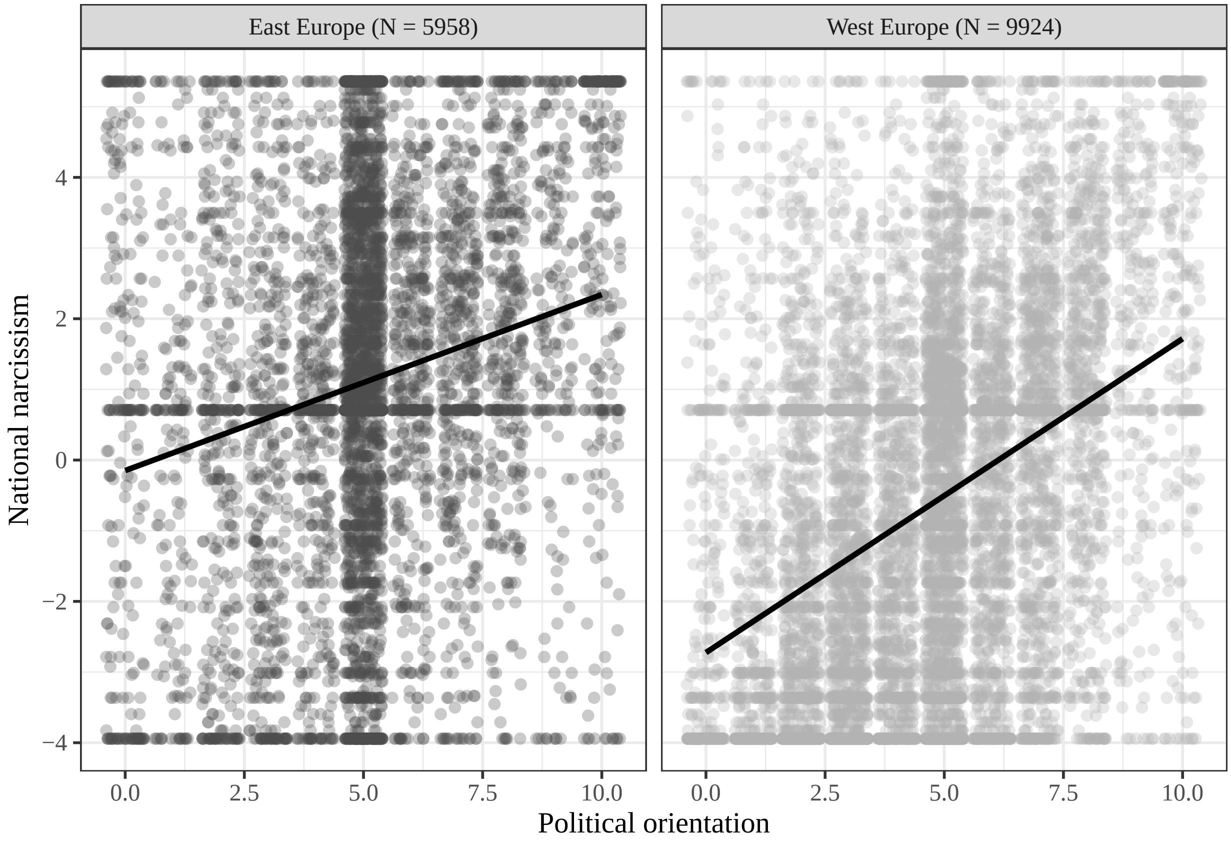


```r
#quadratic
ggplot(df2, aes(x = PoliticId, y = cnar, color = comm)) + geom_jitter(alpha = .3) + stat_smooth(method =
```

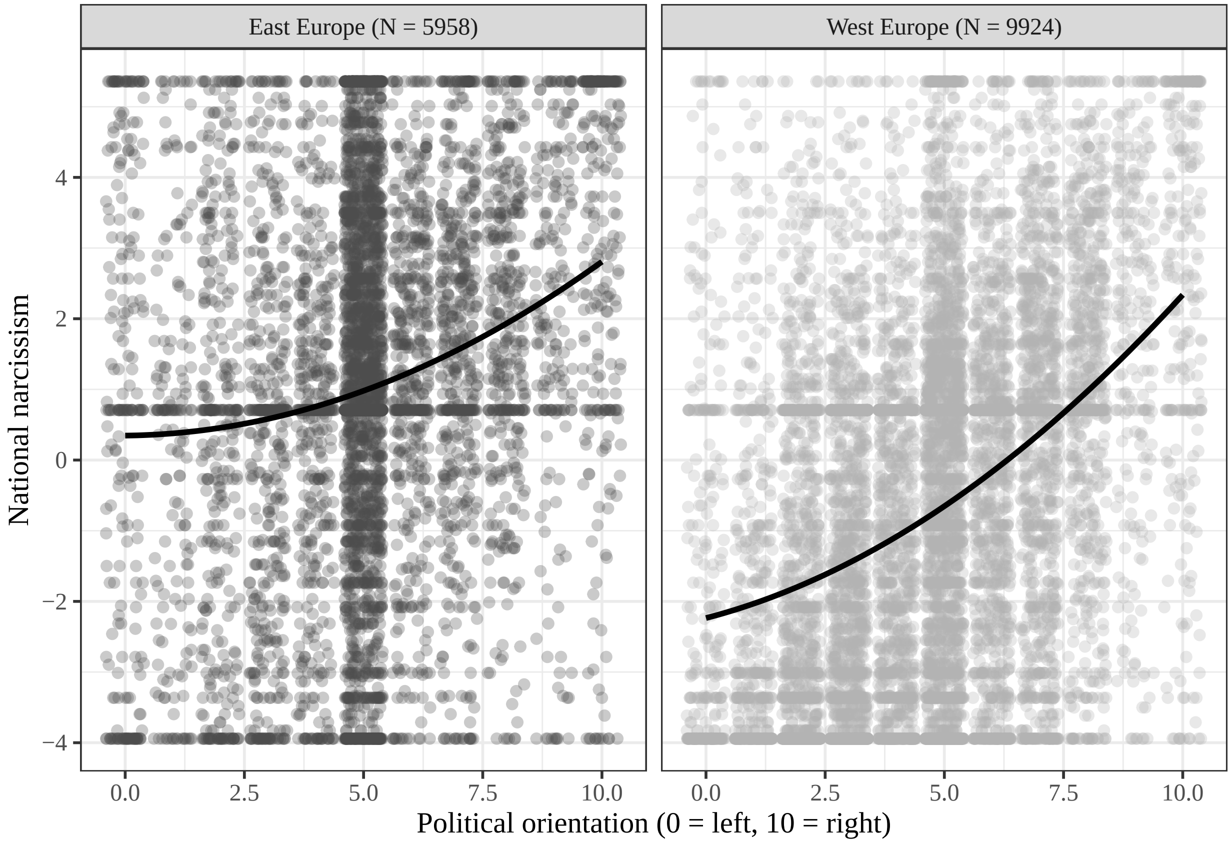


```r
#quadratic
ggplot(df2, aes(x = PoliticId, y = cnar, color = comm)) + stat_smooth(method = "lm", col = "black", for
```

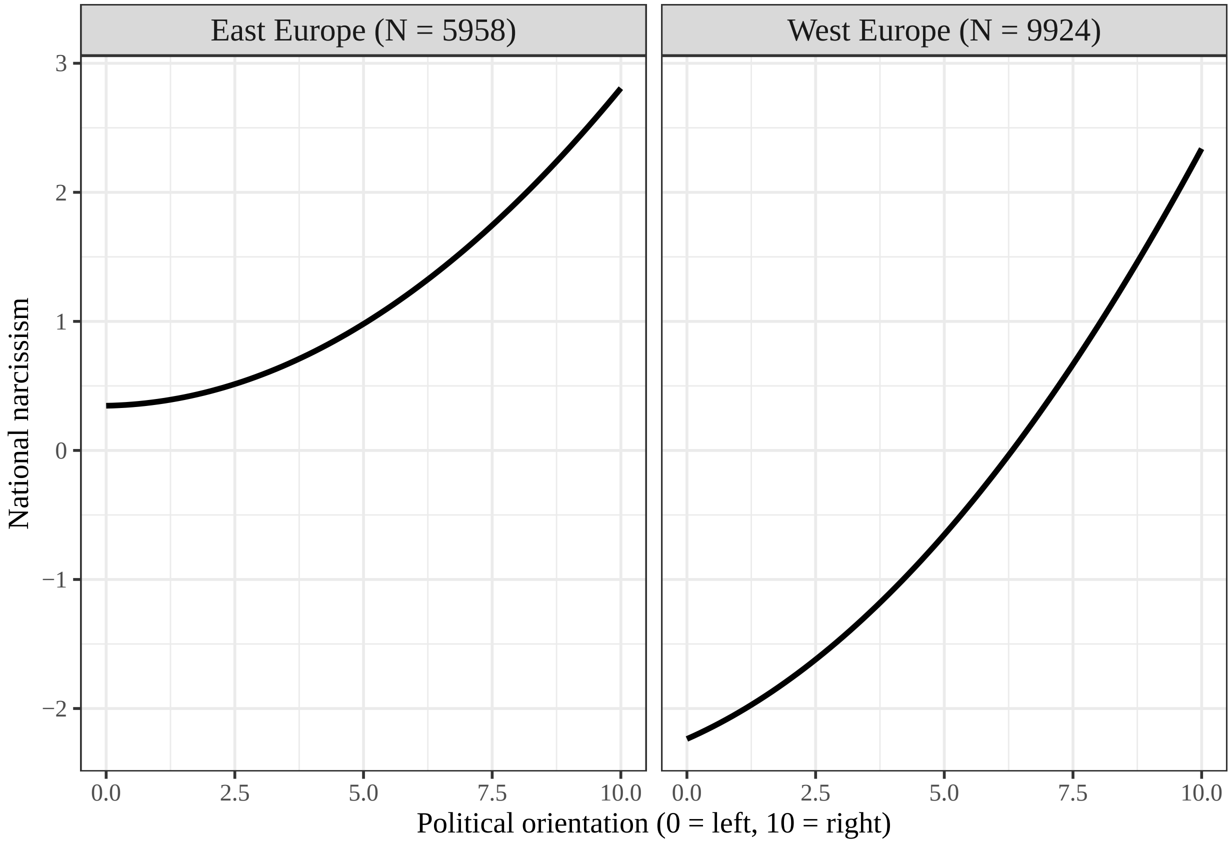


```r
spinburst(modmods)
```

```
## Homogeneous subsets according to significance testing:cnar predicted by age

## [1] "West Europe (N = 9924)" "East Europe (N = 5958)"

## Table with z-values of testing the differences between slopes (Bonferroni adjusted):cnar predicted by

##                          West Europe (N = 9924) East Europe (N = 5958)
## West Europe (N = 9924)                     <NA>                     no
## East Europe (N = 5958)                                            <NA>

## Original coefficients:cnar predicted by age

##                        group     est     se pvalue    beta
## 8  West Europe (N = 9924)  0.0046 0.0048 0.3382  0.0283
## 52 East Europe (N = 5958) -0.0024 0.0041 0.5675 -0.0144

## Homogeneous subsets according to significance testing:cnar predicted by ladder

## [1] "West Europe (N = 9924)" "East Europe (N = 5958)"

## Table with z-values of testing the differences between slopes (Bonferroni adjusted):cnar predicted by
```

```
##                         West Europe (N = 9924) East Europe (N = 5958)
## West Europe (N = 9924)                   <NA>                     no
## East Europe (N = 5958)                                          <NA>
```

```
## Original coefficients:cnar predicted by ladder
```

```
##                      group    est     se pvalue   beta
## 9  West Europe (N = 9924) 0.1278 0.0491 0.0092 0.0906
## 53 East Europe (N = 5958) 0.0245 0.0552 0.6572 0.0168
```

```
## Homogeneous subsets according to significance testing:cnar predicted by natid
```

```
##    [,1]                     [,2]
## V1 "West Europe (N = 9924)" "  "
## V2 "  "                     "East Europe (N = 5958)"
```

```
## Table with z-values of testing the differences between slopes (Bonferroni adjusted):cnar predicted by
```

```
##                         West Europe (N = 9924) East Europe (N = 5958)
## West Europe (N = 9924)                   <NA>                -2.1782
## East Europe (N = 5958)                                            NA
```

```
## Original coefficients:cnar predicted by natid
```

```
##                      group    est     se pvalue   beta
## 6  West Europe (N = 9924) 0.3350 0.0474      0 0.3507
## 50 East Europe (N = 5958) 0.4679 0.0385      0 0.4180
```

```
## Homogeneous subsets according to significance testing:cnar predicted by polid2
```

```
## [1] "West Europe (N = 9924)" "East Europe (N = 5958)"
```

```
## Table with z-values of testing the differences between slopes (Bonferroni adjusted):cnar predicted by
```

```
##                         West Europe (N = 9924) East Europe (N = 5958)
## West Europe (N = 9924)                   <NA>                     no
## East Europe (N = 5958)                                          <NA>
```

```
## Original coefficients:cnar predicted by polid2
```

```
##                      group    est     se pvalue   beta
## 5  West Europe (N = 9924) 0.1924 0.0543  4e-04 0.0942
## 49 East Europe (N = 5958) 0.1381 0.0371  2e-04 0.0783
```

```
## Homogeneous subsets according to significance testing:cnar predicted by PoliticId
```

```
##    [,1]                     [,2]
## V1 "West Europe (N = 9924)" "  "
## V2 "  "                     "East Europe (N = 5958)"
```

```
## Table with z-values of testing the differences between slopes (Bonferroni adjusted):cnar predicted by

##                         West Europe (N = 9924) East Europe (N = 5958)
## West Europe (N = 9924)                   <NA>                 2.4134
## East Europe (N = 5958)                                            NA

## Original coefficients:cnar predicted by PoliticId

##                      group    est     se pvalue   beta
## 4  West Europe (N = 9924) 0.3436 0.0386 0.0000 0.3058
## 48 East Europe (N = 5958) 0.1670 0.0621 0.0072 0.1445

## Homogeneous subsets according to significance testing:cnar predicted by sex1

##    [,1]                     [,2]
## V1 "West Europe (N = 9924)" "  "
## V2 "  "                     "East Europe (N = 5958)"

## Table with z-values of testing the differences between slopes (Bonferroni adjusted):cnar predicted by

##                         West Europe (N = 9924) East Europe (N = 5958)
## West Europe (N = 9924)                   <NA>                -2.2345
## East Europe (N = 5958)                                            NA

## Original coefficients:cnar predicted by sex1

##                      group     est     se pvalue    beta
## 7  West Europe (N = 9924) -0.2297 0.1101 0.0370 -0.0446
## 51 East Europe (N = 5958)  0.0434 0.0530 0.4129  0.0085
```

Robustness test

```r
fi <- read_csv("forimputations.csv")
dim(fi) #16 144
fi$rmiss <- rowSums(is.na(fi))
fi <- subset(fi, rmiss < 3)
dim(fi) #16 099

dat <- mice(fi, m = 1, maxit = 100, method = "pmm")
w <- complete(dat, m = 1)[[1]]
colSums(is.na(w))
write.csv(w, "pmmimputed.csv")
```

```r
c19 <- read_csv("pmmimputed.csv")
```

```
## New names:
## Rows: 16097 Columns: 16
## -- Column specification
## -------------------------------------------------------- Delimiter: "," chr
```

```
## (2): ISO3, comm dbl (14): ...1, ...2, att_check_nobots, revision_coding, natid,
## cnarc1, cnar...
## i Use `spec()` to retrieve the full column specification for this data. i
## Specify the column types or set `show_col_types = FALSE` to quiet this message.
## * `` -> `...1`
## * `...1` -> `...2`
```

```
c19$comm <- ifelse(c19$ISO3 %in% c("BGR", "HRV", "HUN", "LVA", "MKD", "POL", "ROU", "RUS", "SRB", "SVK"
c19 <- subset(c19, comm %in% c("East Europe (N = 5996)", "West Europe (N = 10 101)"))
c20 <- c19[,c("ISO3", "comm", "natid", "cnarc1", "cnarc2", "cnarc3", "political_ideology", "sex1", "age
c20$polid2 <- scale(c20$political_ideology)*scale(c20$political_ideology)
c20$PoliticId <- c20$political_ideology

describe(c20)
```

```
##                    vars     n  mean    sd median trimmed   mad   min   max
## ISO3*                 1 16097  8.59  5.09   9.00    8.54  7.41  1.00  17.0
## comm*                 2 16097  1.63  0.48   2.00    1.66  0.00  1.00   2.0
## natid                 3 16097  7.44  2.64   8.00    7.83  2.97  0.00  10.0
## cnarc1                4 16097  4.18  3.22   5.00    4.00  4.45  0.00  10.0
## cnarc2                5 16097  4.81  3.01   5.00    4.78  2.97  0.00  10.0
## cnarc3                6 16097  4.14  3.21   5.00    3.95  4.45  0.00  10.0
## political_ideology    7 16097  4.79  2.28   5.00    4.77  2.97  0.00  10.0
## sex1                  8 16097  1.51  0.50   2.00    1.51  0.00  1.00   3.0
## age                   9 16097 46.66 15.94  47.00   46.58 19.27 18.00 100.0
## ladder               10 16097  5.48  1.85   6.00    5.42  1.48  1.00  11.0
## polid2               11 16097  1.00  1.34   0.61    0.71  0.90  0.01   5.2
## PoliticId            12 16097  4.79  2.28   5.00    4.77  2.97  0.00  10.0
##                    range  skew kurtosis   se
## ISO3*               16.0  0.01    -1.37 0.04
## comm*                1.0 -0.53    -1.72 0.00
## natid               10.0 -1.01     0.27 0.02
## cnarc1              10.0  0.17    -1.04 0.03
## cnarc2              10.0 -0.10    -0.83 0.02
## cnarc3              10.0  0.18    -1.05 0.03
## political_ideology  10.0  0.06    -0.20 0.02
## sex1                 2.0  0.01    -1.89 0.00
## age                 82.0  0.06    -0.97 0.13
## ladder              10.0  0.28     0.30 0.01
## polid2               5.2  1.68     2.09 0.01
## PoliticId           10.0  0.06    -0.20 0.02
```

```
table(c20$comm)
```

```
##
##   East Europe (N = 5996) West Europe (N = 10 101)
##                     5996                     10101
```

```
table(c20$comm, c20$ISO3)
```

```
##
##                               AUT  CHE  DEU  DNK  ESP  FRA  GBR  HRV  HUN  ITA
```

```
##   East Europe (N = 5996)      0    0    0    0    0    0    0  511  506    0
##   West Europe (N = 10 101) 1373 1053 1587  553 1086 1114  547    0    0  973
##
##                             LVA  NLD  NOR  POL  RUS  SVK  UKR
##   East Europe (N = 5996)    998    0    0 1800  507 1097  577
##   West Europe (N = 10 101)    0 1289  526    0    0    0    0
```

```
model <- 'cnar =~ cnarc1 + cnarc2 + cnarc3
          cnar ~ PoliticId + polid2 + natid + sex1 + age + ladder'

modmodc <- sem(model, estimator = "MLR", data = c20, group = "comm", cluster = "ISO3")
```

```
## Warning in lav_model_vcov(lavmodel = lavmodel, lavsamplestats = lavsamplestats, : lavaan WARNING:
##     The variance-covariance matrix of the estimated parameters (vcov)
##     does not appear to be positive definite! The smallest eigenvalue
##     (= -3.040027e-14) is smaller than zero. This may be a symptom that
##     the model is not identified.
```

```
modmodw <- sem(model, estimator = "MLR", data = c20, group = "comm", group.equal = "loadings", cluster =
```

```
## Warning in lav_model_vcov(lavmodel = lavmodel, lavsamplestats = lavsamplestats, : lavaan WARNING:
##     The variance-covariance matrix of the estimated parameters (vcov)
##     does not appear to be positive definite! The smallest eigenvalue
##     (= -5.377697e-16) is smaller than zero. This may be a symptom that
##     the model is not identified.
```

```
modmods <- sem(model, estimator = "MLR", data = c20, group = "comm", group.equal = c("loadings", "interc
```

```
## Warning in lav_model_vcov(lavmodel = lavmodel, lavsamplestats = lavsamplestats, : lavaan WARNING:
##     The variance-covariance matrix of the estimated parameters (vcov)
##     does not appear to be positive definite! The smallest eigenvalue
##     (= -9.867112e-17) is smaller than zero. This may be a symptom that
##     the model is not identified.
```

```
modmodr <- sem(model, estimator = "MLR", data = c20, group = "comm", group.equal = c("loadings", "interc
```

```
## Warning in lav_model_vcov(lavmodel = lavmodel, lavsamplestats = lavsamplestats, : lavaan WARNING:
##     The variance-covariance matrix of the estimated parameters (vcov)
##     does not appear to be positive definite! The smallest eigenvalue
##     (= 1.953575e-16) is close to zero. This may be a symptom that the
##     model is not identified.
```

```
summary(compareFit(modmodc, modmodw, modmods, modmodr, nested = T), fit.measures = c("cfi", "cfi.robust
```

```
## ################### Nested Model Comparison #########################
##
## Scaled Chi-Squared Difference Test (method = "satorra.bentler.2001")
##
## lavaan NOTE:
##     The "Chisq" column contains standard test statistics, not the
```

```
##     robust test that should be reported per model. A robust difference
##     test is a function of two standard (not robust) statistics.
##
##         Df    AIC    BIC  Chisq Chisq diff Df diff Pr(>Chisq)
## modmodc 24 214199 214429 211.96
## modmodw 26 214214 214429 231.23     4.1129       2  0.1279070
## modmods 28 214242 214442 263.11     0.7662       2  0.6817445
## modmodr 34 214403 214557 436.31    26.5775       6  0.0001737 ***
## ---
## Signif. codes:  0 '***' 0.001 '**' 0.01 '*' 0.05 '.' 0.1 ' ' 1
##
## ####################### Model Fit Indices ###########################
##           cfi cfi.robust rmsea.robust  srmr
## modmodc .994†         NA         .029  .008†
## modmodw .993          NA         .029  .009
## modmods .992          NA         .023† .010
## modmodr .986          NA         .031  .020
##
## ################## Differences in Fit Indices #######################
##                     cfi cfi.robust rmsea.robust  srmr
## modmodw - modmodc -0.001         NA        0.000 0.001
## modmods - modmodw -0.001         NA       -0.006 0.001
## modmodr - modmods -0.006         NA        0.007 0.010
```

```r
model <- 'cnar =~ cnarc1 + cnarc2 + cnarc3'
modmod <- sem(model, estimator = "MLR", data = c20)

df <- as.data.frame(estimate_lavaan_ten_berge(modmod)$scores)
df2 <- cbind(c20, df)

#overall
ggplot(df2, aes(x = PoliticId, y = cnar)) + geom_jitter(alpha = .2, col = "gray50") + stat_smooth(metho
```

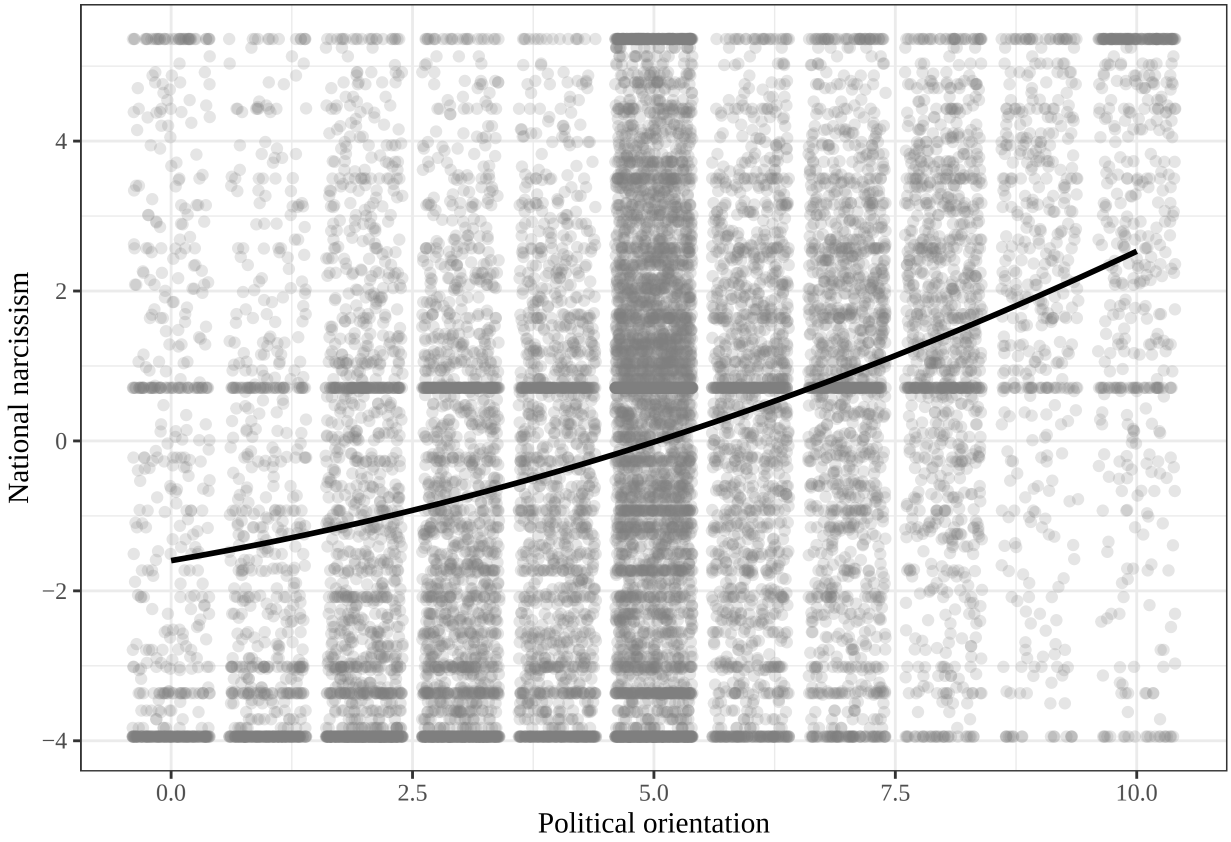


```
#linear
ggplot(df2, aes(x = PoliticId, y = cnar, color = comm)) + geom_jitter(alpha = .3) + geom_smooth(method =
```

```
## `geom_smooth()` using formula = 'y ~ x'
```

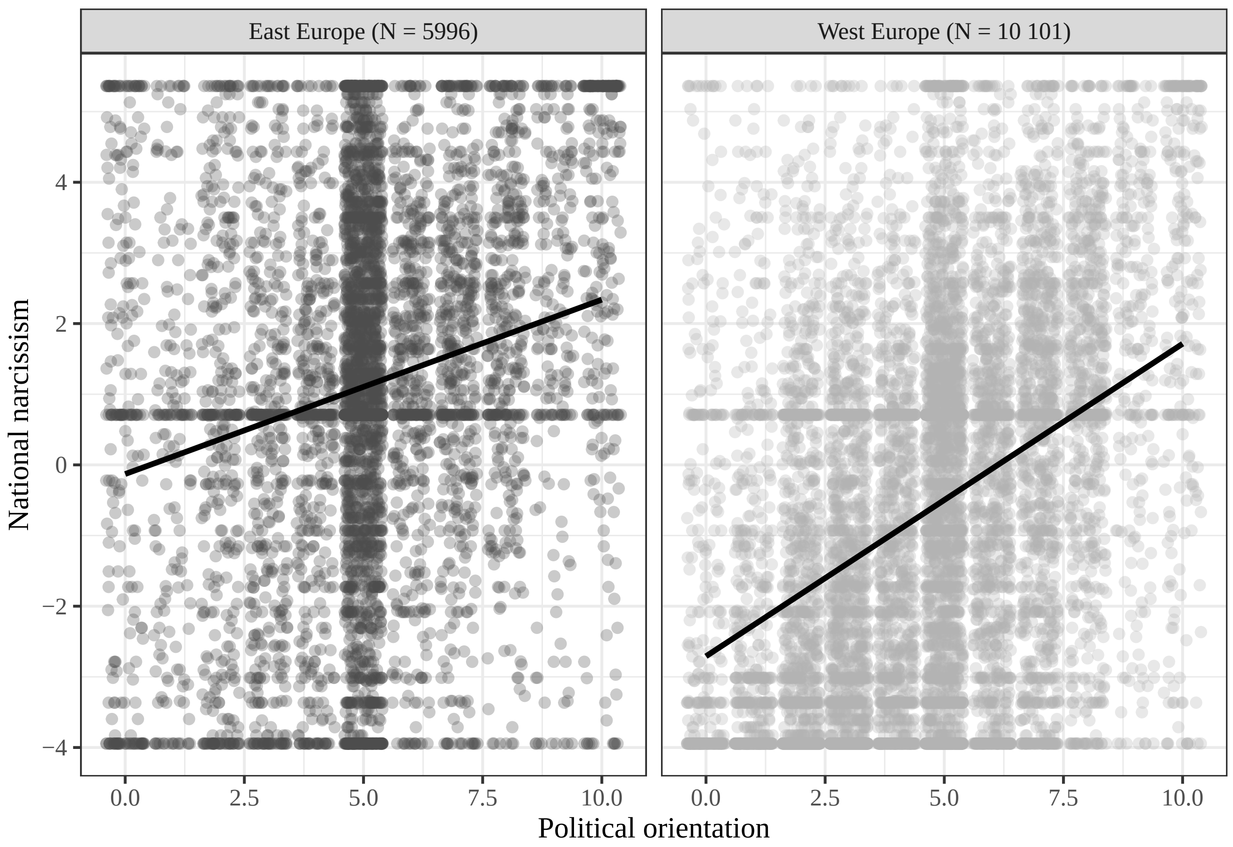


```
#quadratic
ggplot(df2, aes(x = PoliticId, y = cnar, color = comm)) + geom_jitter(alpha = .3) + stat_smooth(method =
```

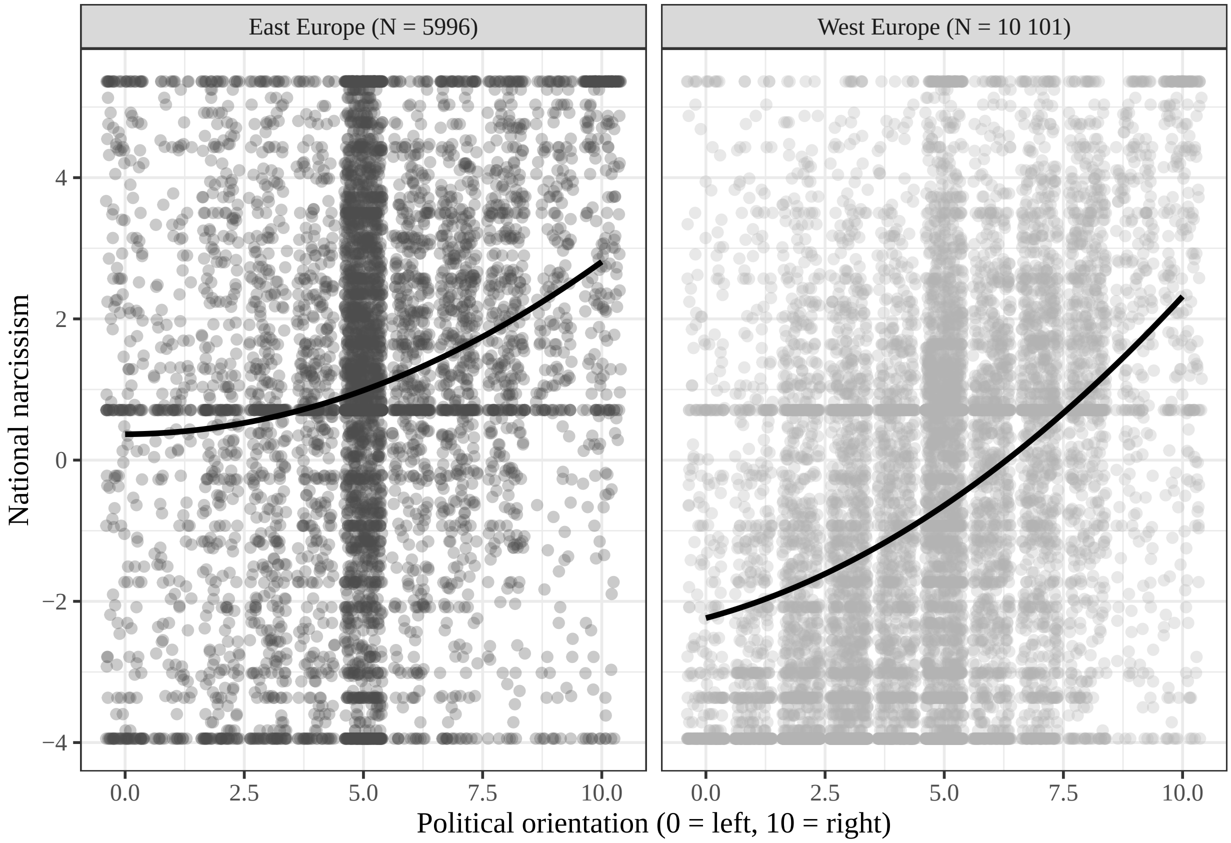


```
#quadratic
ggplot(df2, aes(x = PoliticId, y = cnar, color = comm)) + stat_smooth(method = "lm", col = "black", for
```

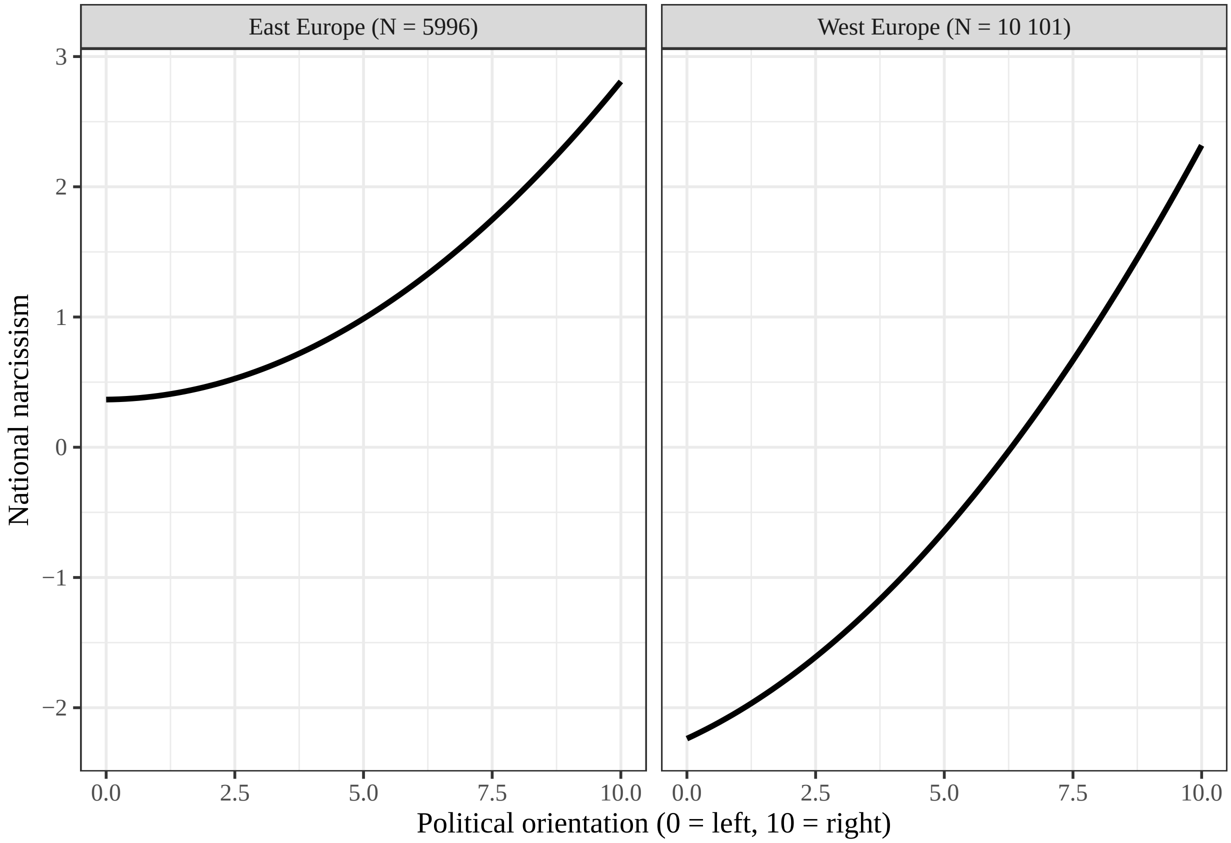


```r
spinburst(modmods)
```

```
## Homogeneous subsets according to significance testing:cnar predicted by age

## [1] "West Europe (N = 10 101)" "East Europe (N = 5996)"

## Table with z-values of testing the differences between slopes (Bonferroni adjusted):cnar predicted by

##                          West Europe (N = 10 101) East Europe (N = 5996)
## West Europe (N = 10 101)                     <NA>                     no
## East Europe (N = 5996)                                              <NA>

## Original coefficients:cnar predicted by age

##                         group     est     se pvalue    beta
## 8  West Europe (N = 10 101)  0.0048 0.0046 0.2963  0.0299
## 52   East Europe (N = 5996) -0.0024 0.0041 0.5519 -0.0148

## Homogeneous subsets according to significance testing:cnar predicted by ladder

## [1] "West Europe (N = 10 101)" "East Europe (N = 5996)"

## Table with z-values of testing the differences between slopes (Bonferroni adjusted):cnar predicted by
```

```
##                          West Europe (N = 10 101) East Europe (N = 5996)
## West Europe (N = 10 101)                     <NA>                     no
## East Europe (N = 5996)                                              <NA>
```

```
## Original coefficients:cnar predicted by ladder
```

```
##                       group    est     se pvalue   beta
## 9  West Europe (N = 10 101) 0.1274 0.0488 0.0091 0.0903
## 53   East Europe (N = 5996) 0.0249 0.0551 0.6515 0.0171
```

```
## Homogeneous subsets according to significance testing:cnar predicted by natid
```

```
##    [,1]                       [,2]
## V1 "West Europe (N = 10 101)" "  "
## V2 "  "                       "East Europe (N = 5996)"
```

```
## Table with z-values of testing the differences between slopes (Bonferroni adjusted):cnar predicted by
```

```
##                          West Europe (N = 10 101) East Europe (N = 5996)
## West Europe (N = 10 101)                     <NA>                -2.2023
## East Europe (N = 5996)                                                NA
```

```
## Original coefficients:cnar predicted by natid
```

```
##                       group    est     se pvalue   beta
## 6  West Europe (N = 10 101) 0.3331 0.0474      0 0.3482
## 50   East Europe (N = 5996) 0.4674 0.0384      0 0.4175
```

```
## Homogeneous subsets according to significance testing:cnar predicted by polid2
```

```
## [1] "West Europe (N = 10 101)" "East Europe (N = 5996)"
```

```
## Table with z-values of testing the differences between slopes (Bonferroni adjusted):cnar predicted by
```

```
##                          West Europe (N = 10 101) East Europe (N = 5996)
## West Europe (N = 10 101)                     <NA>                     no
## East Europe (N = 5996)                                              <NA>
```

```
## Original coefficients:cnar predicted by polid2
```

```
##                       group    est     se pvalue   beta
## 5  West Europe (N = 10 101) 0.1870 0.0522  3e-04 0.0916
## 49   East Europe (N = 5996) 0.1372 0.0374  2e-04 0.0779
```

```
## Homogeneous subsets according to significance testing:cnar predicted by PoliticId
```

```
##    [,1]                       [,2]
## V1 "West Europe (N = 10 101)" "  "
## V2 "  "                       "East Europe (N = 5996)"
```

```
## Table with z-values of testing the differences between slopes (Bonferroni adjusted):cnar predicted by

##                          West Europe (N = 10 101) East Europe (N = 5996)
## West Europe (N = 10 101)                     <NA>                 2.3999
## East Europe (N = 5996)                                                NA

## Original coefficients:cnar predicted by PoliticId

##                         group    est     se pvalue   beta
## 4  West Europe (N = 10 101) 0.3432 0.0397 0.0000 0.3054
## 48   East Europe (N = 5996) 0.1657 0.0624 0.0079 0.1435

## Homogeneous subsets according to significance testing:cnar predicted by sex1

##    [,1]                       [,2]
## V1 "West Europe (N = 10 101)" "  "
## V2 "  "                       "East Europe (N = 5996)"

## Table with z-values of testing the differences between slopes (Bonferroni adjusted):cnar predicted by

##                          West Europe (N = 10 101) East Europe (N = 5996)
## West Europe (N = 10 101)                     <NA>                -2.2865
## East Europe (N = 5996)                                                NA

## Original coefficients:cnar predicted by sex1

##                         group     est     se pvalue    beta
## 7  West Europe (N = 10 101) -0.2340 0.1114 0.0358 -0.0454
## 51   East Europe (N = 5996)  0.0473 0.0521 0.3640  0.0093
```

Robustness test 2

```
c22 <- split.data.frame(c20, c20$ISO3)
for(i in seq_along(c22)){
  c22[[i]]$polid2_2 <- scale(c22[[i]]$political_ideology)*scale(c22[[i]]$political_ideology)
}
c23 <- bind_rows(c22)
model <- 'cnar =~ cnarc1 + cnarc2 + cnarc3
          cnar ~ PoliticId + polid2_2 + natid + sex1 + age + ladder'

modmodc <- sem(model, estimator = "MLR", data = c23, group = "comm", cluster = "ISO3")
```

```
## Warning in lav_model_vcov(lavmodel = lavmodel, lavsamplestats = lavsamplestats, : lavaan WARNING:
##     The variance-covariance matrix of the estimated parameters (vcov)
##     does not appear to be positive definite! The smallest eigenvalue
##     (= -3.278761e-14) is smaller than zero. This may be a symptom that
##     the model is not identified.
```

```r
modmodw <- sem(model, estimator = "MLR", data = c23, group = "comm", group.equal = "loadings", cluster =
```

```
## Warning in lav_model_vcov(lavmodel = lavmodel, lavsamplestats = lavsamplestats, : lavaan WARNING:
##     The variance-covariance matrix of the estimated parameters (vcov)
##     does not appear to be positive definite! The smallest eigenvalue
##     (= -6.814089e-17) is smaller than zero. This may be a symptom that
##     the model is not identified.
```

```r
modmods <- sem(model, estimator = "MLR", data = c23, group = "comm", group.equal = c("loadings", "inter
```

```
## Warning in lav_model_vcov(lavmodel = lavmodel, lavsamplestats = lavsamplestats, : lavaan WARNING:
##     The variance-covariance matrix of the estimated parameters (vcov)
##     does not appear to be positive definite! The smallest eigenvalue
##     (= -4.688796e-17) is smaller than zero. This may be a symptom that
##     the model is not identified.
```

```r
modmodr <- sem(model, estimator = "MLR", data = c23, group = "comm", group.equal = c("loadings", "inter
```

```
## Warning in lav_model_vcov(lavmodel = lavmodel, lavsamplestats = lavsamplestats, : lavaan WARNING:
##     The variance-covariance matrix of the estimated parameters (vcov)
##     does not appear to be positive definite! The smallest eigenvalue
##     (= 2.644837e-16) is close to zero. This may be a symptom that the
##     model is not identified.
```

```r
summary(compareFit(modmodc, modmodw, modmods, modmodr, nested = T), fit.measures = c("cfi", "cfi.robust
```

```
## ################### Nested Model Comparison #########################
##
## Scaled Chi-Squared Difference Test (method = "satorra.bentler.2001")
##
## lavaan NOTE:
##     The "Chisq" column contains standard test statistics, not the
##     robust test that should be reported per model. A robust difference
##     test is a function of two standard (not robust) statistics.
##
##         Df    AIC    BIC  Chisq Chisq diff Df diff Pr(>Chisq)
## modmodc 24 214222 214452 205.38
## modmodw 26 214237 214452 224.87     4.2593       2  0.1188770
## modmods 28 214265 214465 256.70     0.7634       2  0.6827031
## modmodr 34 214406 214560 409.77    23.0879       6  0.0007676 ***
## ---
## Signif. codes:  0 '***' 0.001 '**' 0.01 '*' 0.05 '.' 0.1 ' ' 1
##
## ####################### Model Fit Indices ###########################
##           cfi cfi.robust rmsea.robust  srmr
## modmodc .994†        NA         .028  .008†
## modmodw .993         NA         .028  .009
## modmods .992         NA         .022† .010
## modmodr .987         NA         .029  .019
##
## ################## Differences in Fit Indices #######################
```

```
##                        cfi cfi.robust rmsea.robust  srmr
## modmodw - modmodc -0.001         NA        0.000 0.001
## modmods - modmodw -0.001         NA       -0.006 0.001
## modmodr - modmods -0.005         NA        0.006 0.008
```

```r
model <- 'cnar =~ cnarc1 + cnarc2 + cnarc3'
modmod <- sem(model, estimator = "MLR", data = c23)

df <- as.data.frame(estimate_lavaan_ten_berge(modmod)$scores)
df2 <- cbind(c23, df)

#overall
ggplot(df2, aes(x = PoliticId, y = cnar)) + geom_jitter(alpha = .2, col = "gray50") + stat_smooth(metho
```

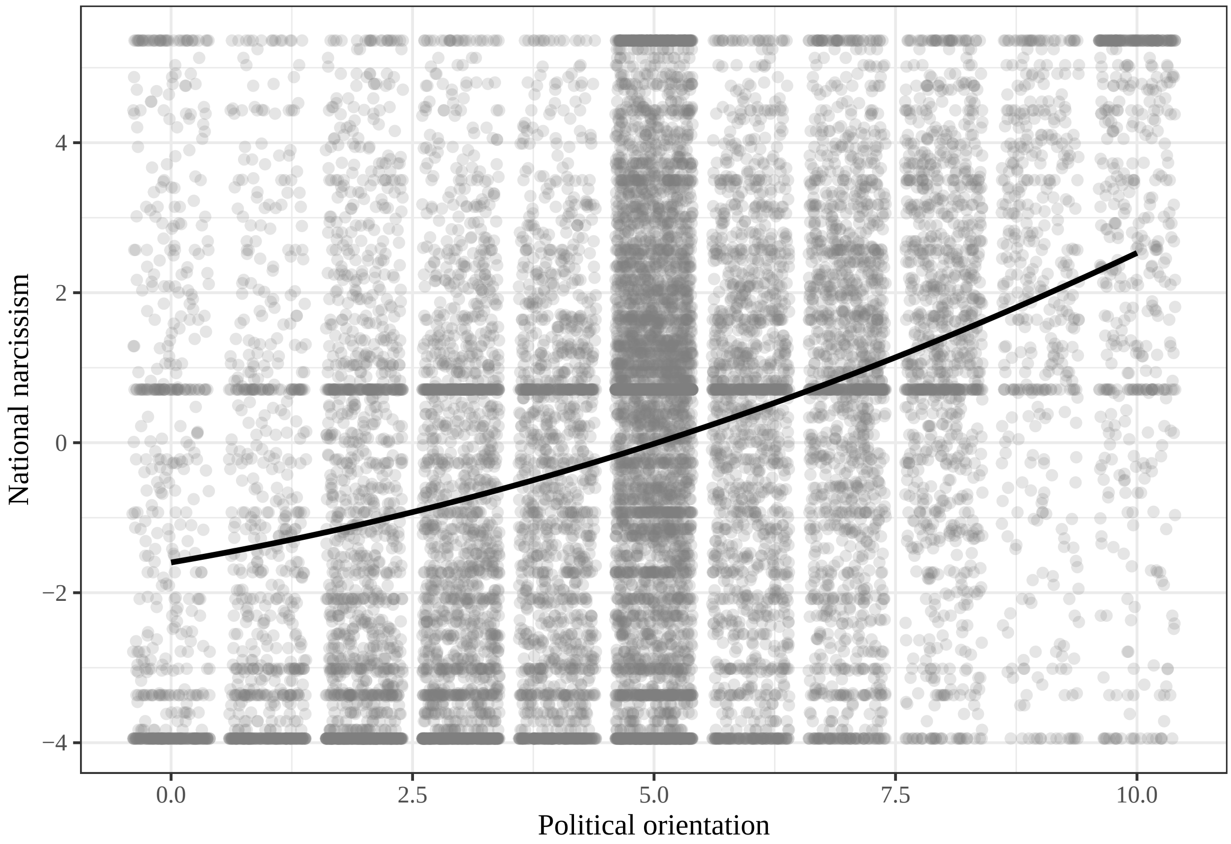


```r
#linear
ggplot(df2, aes(x = PoliticId, y = cnar, color = comm)) + geom_jitter(alpha = .3) + geom_smooth(method =
```

```
## `geom_smooth()` using formula = 'y ~ x'
```

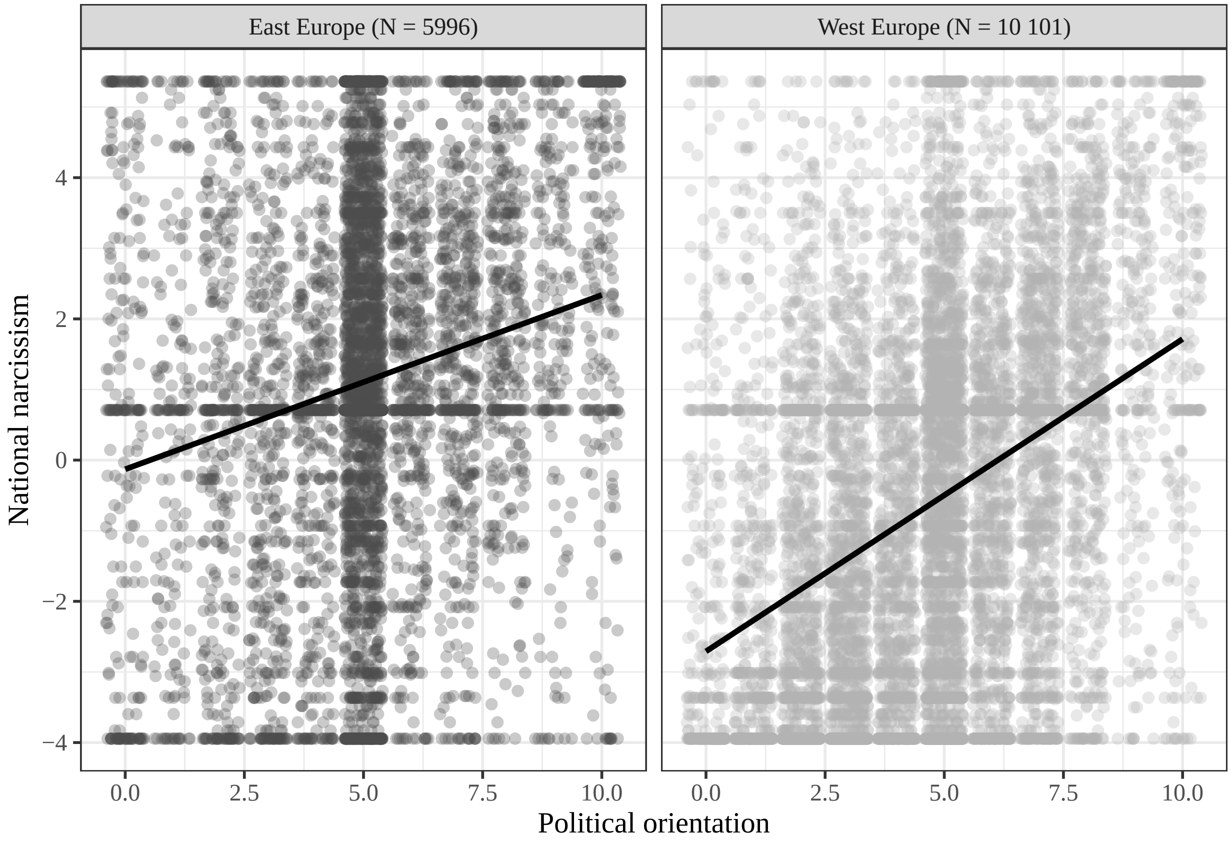


```
#quadratic
ggplot(df2, aes(x = PoliticId, y = cnar, color = comm)) + geom_jitter(alpha = .3) + stat_smooth(method =
```

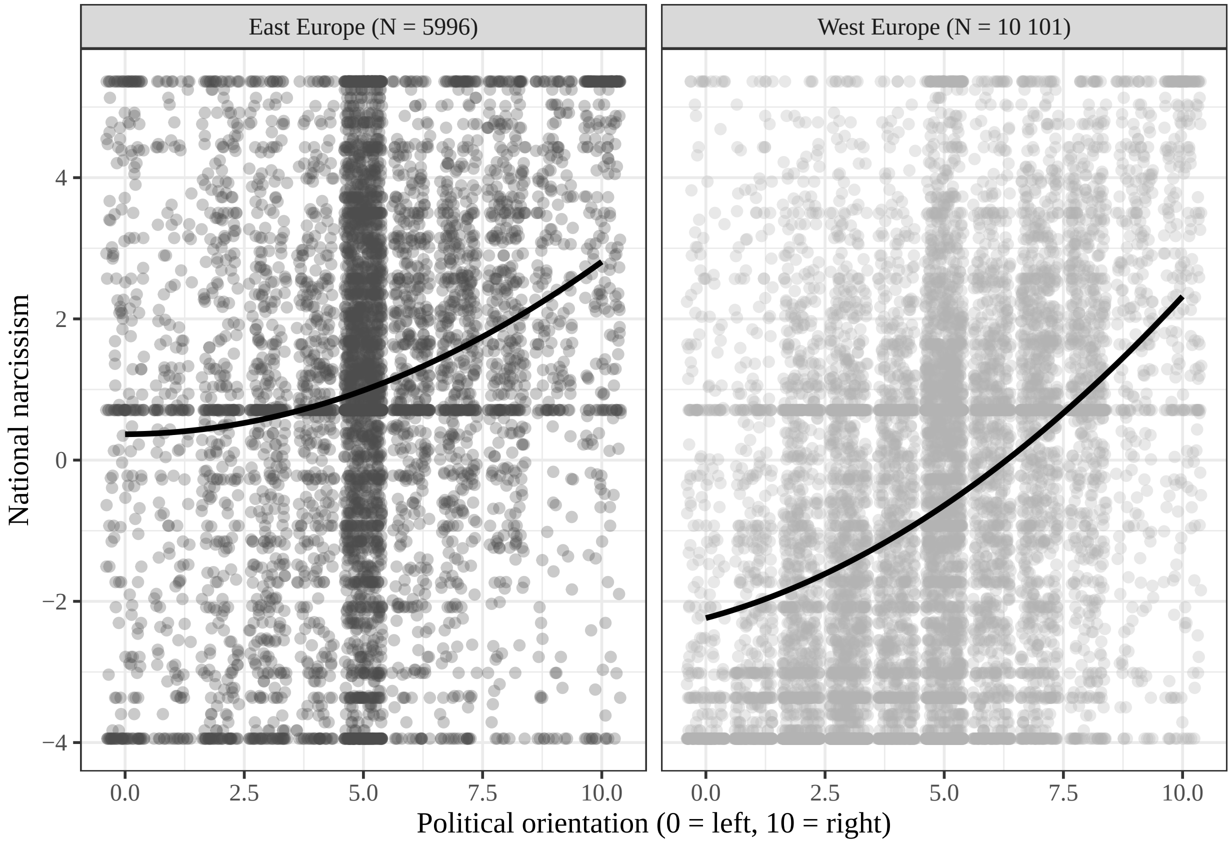


```
#quadratic
ggplot(df2, aes(x = PoliticId, y = cnar, color = comm)) + stat_smooth(method = "lm", col = "black", for
```

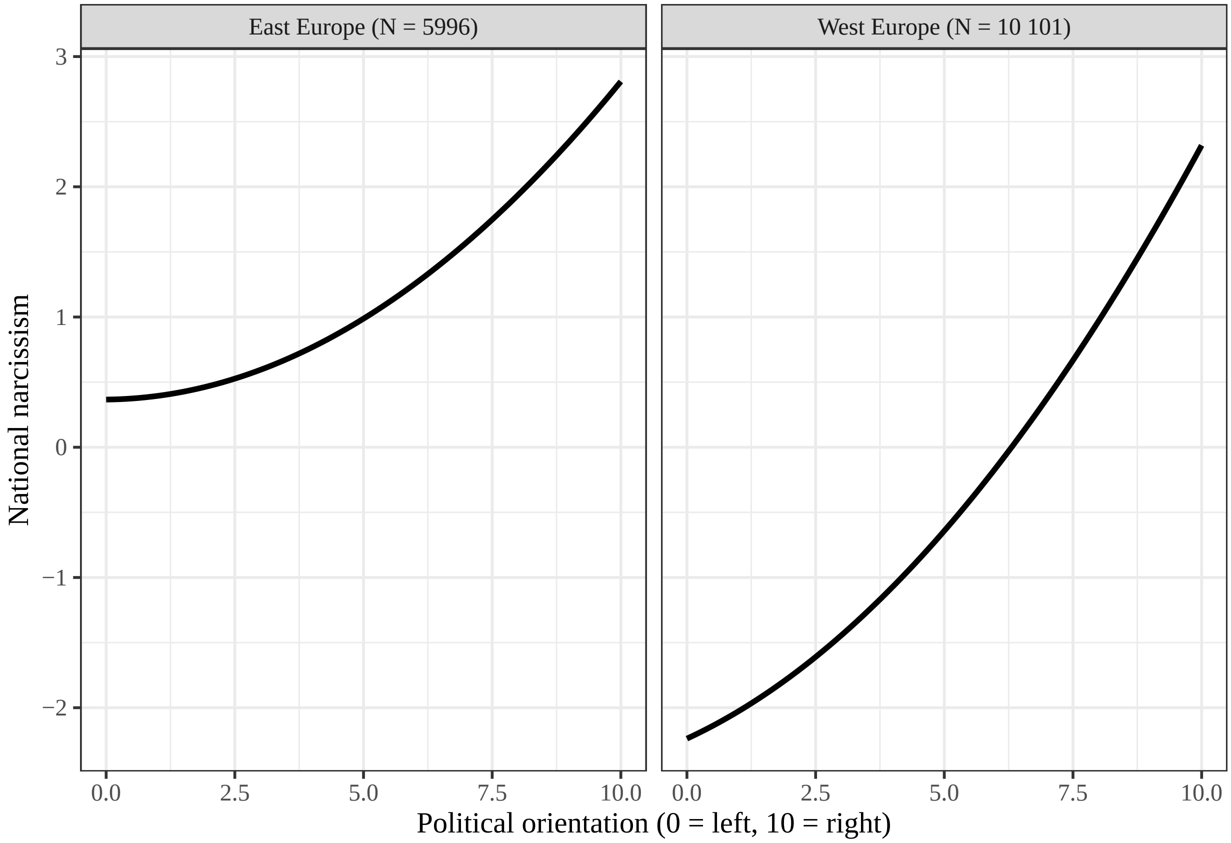


```r
spinburst(modmods)
```

```
## Homogeneous subsets according to significance testing:cnar predicted by age

## [1] "West Europe (N = 10 101)" "East Europe (N = 5996)"

## Table with z-values of testing the differences between slopes (Bonferroni adjusted):cnar predicted by

##                          West Europe (N = 10 101) East Europe (N = 5996)
## West Europe (N = 10 101)                     <NA>                     no
## East Europe (N = 5996)                                              <NA>

## Original coefficients:cnar predicted by age

##                       group     est     se pvalue    beta
## 8  West Europe (N = 10 101)  0.0045 0.0047 0.3343  0.0279
## 52   East Europe (N = 5996) -0.0024 0.0040 0.5483 -0.0147

## Homogeneous subsets according to significance testing:cnar predicted by ladder

## [1] "West Europe (N = 10 101)" "East Europe (N = 5996)"

## Table with z-values of testing the differences between slopes (Bonferroni adjusted):cnar predicted by
```

```
##                           West Europe (N = 10 101) East Europe (N = 5996)
## West Europe (N = 10 101)                     <NA>                     no
## East Europe (N = 5996)                                              <NA>
```

```
## Original coefficients:cnar predicted by ladder
```

```
##                        group    est     se pvalue   beta
## 9  West Europe (N = 10 101) 0.1258 0.0507 0.0132 0.0892
## 53   East Europe (N = 5996) 0.0257 0.0552 0.6417 0.0176
```

```
## Homogeneous subsets according to significance testing:cnar predicted by natid
```

```
##    [,1]                       [,2]
## V1 "West Europe (N = 10 101)" "  "
## V2 "  "                       "East Europe (N = 5996)"
```

```
## Table with z-values of testing the differences between slopes (Bonferroni adjusted):cnar predicted by
```

```
##                           West Europe (N = 10 101) East Europe (N = 5996)
## West Europe (N = 10 101)                     <NA>                -2.3433
## East Europe (N = 5996)                                                NA
```

```
## Original coefficients:cnar predicted by natid
```

```
##                        group    est     se pvalue   beta
## 6  West Europe (N = 10 101) 0.3279 0.0466      0 0.3428
## 50   East Europe (N = 5996) 0.4688 0.0380      0 0.4186
```

```
## Homogeneous subsets according to significance testing:cnar predicted by polid2_2
```

```
## [1] "West Europe (N = 10 101)" "East Europe (N = 5996)"
```

```
## Table with z-values of testing the differences between slopes (Bonferroni adjusted):cnar predicted by
```

```
##                           West Europe (N = 10 101) East Europe (N = 5996)
## West Europe (N = 10 101)                     <NA>                     no
## East Europe (N = 5996)                                              <NA>
```

```
## Original coefficients:cnar predicted by polid2_2
```

```
##                        group    est     se pvalue   beta
## 5  West Europe (N = 10 101) 0.1362 0.0220      0 0.0707
## 49   East Europe (N = 5996) 0.1532 0.0323      0 0.0934
```

```
## Homogeneous subsets according to significance testing:cnar predicted by PoliticId
```

```
##    [,1]                       [,2]
## V1 "West Europe (N = 10 101)" "  "
## V2 "  "                       "East Europe (N = 5996)"
```

```
## Table with z-values of testing the differences between slopes (Bonferroni adjusted):cnar predicted by

##                          West Europe (N = 10 101) East Europe (N = 5996)
## West Europe (N = 10 101)                     <NA>                 2.1713
## East Europe (N = 5996)                                                NA
```

```
## Original coefficients:cnar predicted by PoliticId

##                        group    est     se pvalue   beta
## 4  West Europe (N = 10 101) 0.3365 0.0327 0.0000 0.2995
## 48   East Europe (N = 5996) 0.1808 0.0639 0.0046 0.1565
```

```
## Homogeneous subsets according to significance testing:cnar predicted by sex1

##    [,1]                       [,2]
## V1 "West Europe (N = 10 101)" "  "
## V2 "  "                       "East Europe (N = 5996)"
```

```
## Table with z-values of testing the differences between slopes (Bonferroni adjusted):cnar predicted by

##                          West Europe (N = 10 101) East Europe (N = 5996)
## West Europe (N = 10 101)                     <NA>                 -2.377
## East Europe (N = 5996)                                                NA
```

```
## Original coefficients:cnar predicted by sex1

##                        group     est     se pvalue    beta
## 7  West Europe (N = 10 101) -0.2341 0.1095 0.0326 -0.0454
## 51   East Europe (N = 5996)  0.0533 0.0512 0.2981  0.0105
```

Robustness test 3

```r
library(mgcv)
df2$comm <- as.factor(df2$comm)

gamout <- gam(cnar ~ s(PoliticId, by = comm) + natid + sex1 + age + ladder, data = df2, method = "REML")
summary(gamout)
```

```
##
## Family: gaussian
## Link function: identity
##
## Formula:
## cnar ~ s(PoliticId, by = comm) + natid + sex1 + age + ladder
##
## Parametric coefficients:
##               Estimate Std. Error t value Pr(>|t|)
## (Intercept) -3.4524365  0.1070759 -32.243  < 2e-16 ***
## natid        0.3965269  0.0074412  53.288  < 2e-16 ***
```

```
## sex1         -0.1415650  0.0369708  -3.829 0.000129 ***
## age          -0.0000924  0.0011740  -0.079 0.937264
## ladder        0.1300168  0.0100356  12.956  < 2e-16 ***
## ---
## Signif. codes:  0 '***' 0.001 '**' 0.01 '*' 0.05 '.' 0.1 ' ' 1
##
## Approximate significance of smooth terms:
##                                            edf Ref.df      F p-value
## s(PoliticId):commEast Europe (N = 5996)   7.504  8.433  48.13  <2e-16 ***
## s(PoliticId):commWest Europe (N = 10 101) 7.041  8.088 120.05  <2e-16 ***
## ---
## Signif. codes:  0 '***' 0.001 '**' 0.01 '*' 0.05 '.' 0.1 ' ' 1
##
## R-sq.(adj) =  0.276   Deviance explained = 27.7%
## -REML =  36482  Scale est. = 5.4178    n = 16097
```

```r
gam.check(gamout)
```

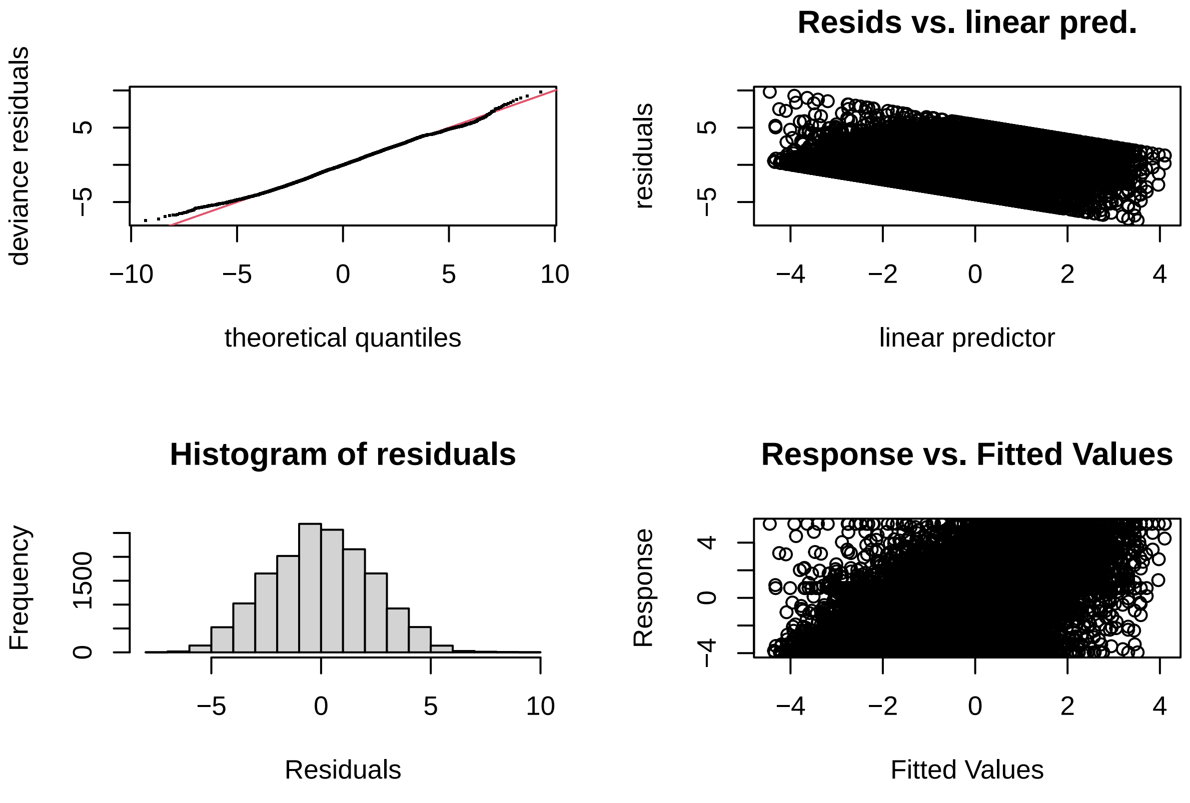


```
##
## Method: REML   Optimizer: outer newton
## full convergence after 6 iterations.
## Gradient range [-1.621724e-05,3.239021e-06]
## (score 36481.98 & scale 5.417763).
## Hessian positive definite, eigenvalue range [2.066062,8045.002].
## Model rank =  23 / 23
```

```
##
## Basis dimension (k) checking results. Low p-value (k-index<1) may
## indicate that k is too low, especially if edf is close to k'.
##
##                                            k'  edf k-index p-value
## s(PoliticId):commEast Europe (N = 5996)   9.00 7.50    0.99    0.28
## s(PoliticId):commWest Europe (N = 10 101) 9.00 7.04    0.99    0.28
```

```r
concurvity(gamout)
```

```
##                para s(PoliticId):commEast Europe (N = 5996)
## worst    0.9707118                               0.06077773
## observed 0.9707118                               0.04192463
## estimate 0.9707118                               0.02152421
##          s(PoliticId):commWest Europe (N = 10 101)
## worst                                   0.09846917
## observed                                0.07850014
## estimate                                0.07121984
```

```r
plot(gamout, pages = 1, xlab = "political ideology", ylab = "collective narcissism")
```

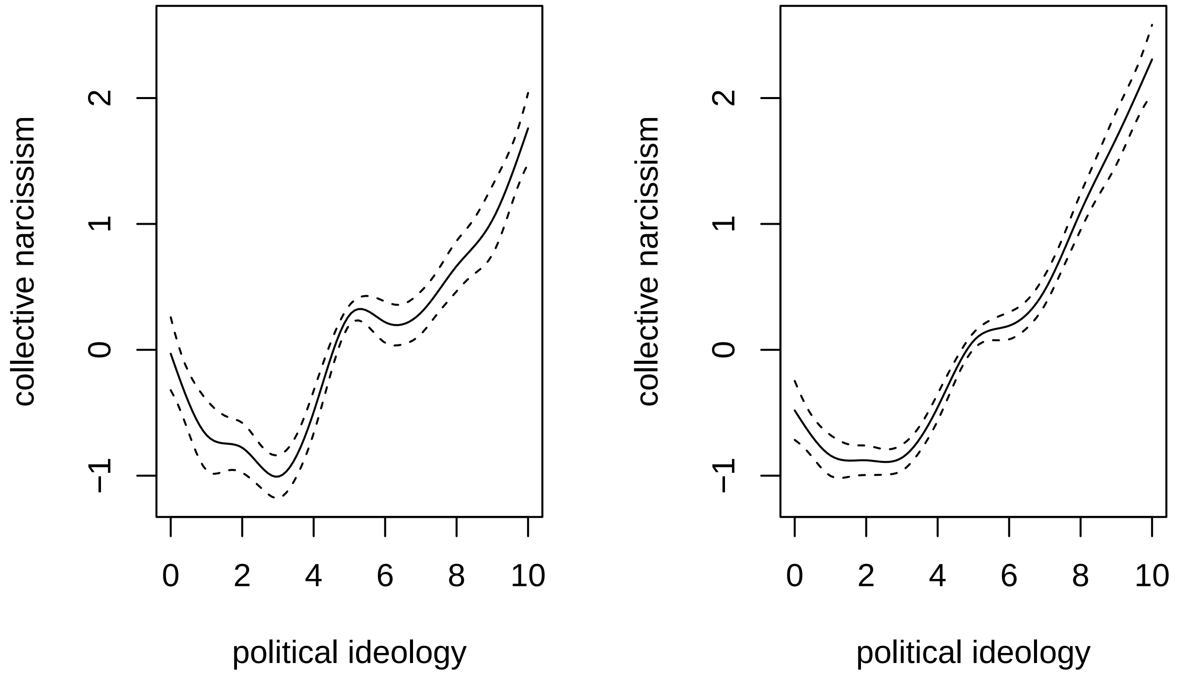


```r
gamout <- gam(cnar ~ s(PoliticId, by = comm, k = 2) + natid + sex1 + age + ladder, data = df2, method =
```

```
## Warning in smooth.construct.tp.smooth.spec(object, dk$data, dk$knots): basis dimension, k, increased
```

```r
summary(gamout)
```

```
##
## Family: gaussian
## Link function: identity
##
## Formula:
## cnar ~ s(PoliticId, by = comm, k = 2) + natid + sex1 + age +
##     ladder
##
## Parametric coefficients:
##              Estimate Std. Error t value Pr(>|t|)
## (Intercept) -3.563517   0.107007 -33.302  < 2e-16 ***
## natid        0.403313   0.007465  54.030  < 2e-16 ***
## sex1        -0.114130   0.037062  -3.079  0.00208 **
## age         -0.001149   0.001177  -0.976  0.32886
## ladder       0.145108   0.010017  14.486  < 2e-16 ***
## ---
## Signif. codes:  0 '***' 0.001 '**' 0.01 '*' 0.05 '.' 0.1 ' ' 1
##
## Approximate significance of smooth terms:
##                                            edf Ref.df     F p-value
## s(PoliticId):commEast Europe (N = 5996)   1.938  1.996 138.7  <2e-16 ***
## s(PoliticId):commWest Europe (N = 10 101) 1.989  2.000 445.4  <2e-16 ***
## ---
## Signif. codes:  0 '***' 0.001 '**' 0.01 '*' 0.05 '.' 0.1 ' ' 1
##
## R-sq.(adj) =  0.267   Deviance explained = 26.8%
## -REML =  36556  Scale est. = 5.4799    n = 16097
```

```r
gam.check(gamout)
```

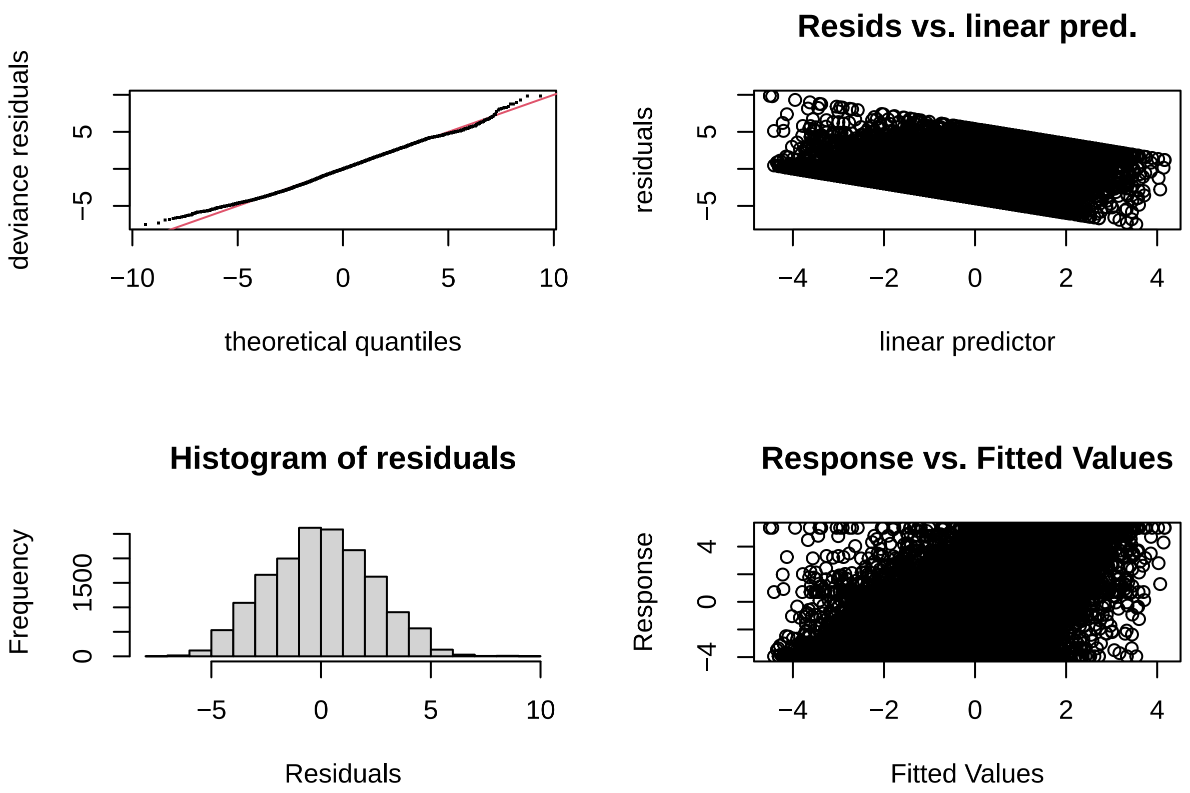


```
##
## Method: REML   Optimizer: outer newton
## full convergence after 5 iterations.
## Gradient range [-0.01950251,0.01364743]
## (score 36556.39 & scale 5.4799).
## Hessian positive definite, eigenvalue range [0.4520069,8045.02].
## Model rank =  9 / 9
##
## Basis dimension (k) checking results. Low p-value (k-index<1) may
## indicate that k is too low, especially if edf is close to k'.
##
##                                              k'  edf k-index p-value
## s(PoliticId):commEast Europe (N = 5996)    2.00 1.94    0.98    0.12
## s(PoliticId):commWest Europe (N = 10 101) 2.00 1.99    0.98    0.11
```

```
concurvity(gamout)
```

```
##                para s(PoliticId):commEast Europe (N = 5996)
## worst    0.970271                              0.02905202
## observed 0.970271                              0.02351187
## estimate 0.970271                              0.02577856
##          s(PoliticId):commWest Europe (N = 10 101)
## worst                                   0.09474677
## observed                                0.07607031
## estimate                                0.08797356
```

```
plot(gamout, pages = 1, xlab = "political ideology", ylab = "collective narcissism")
```

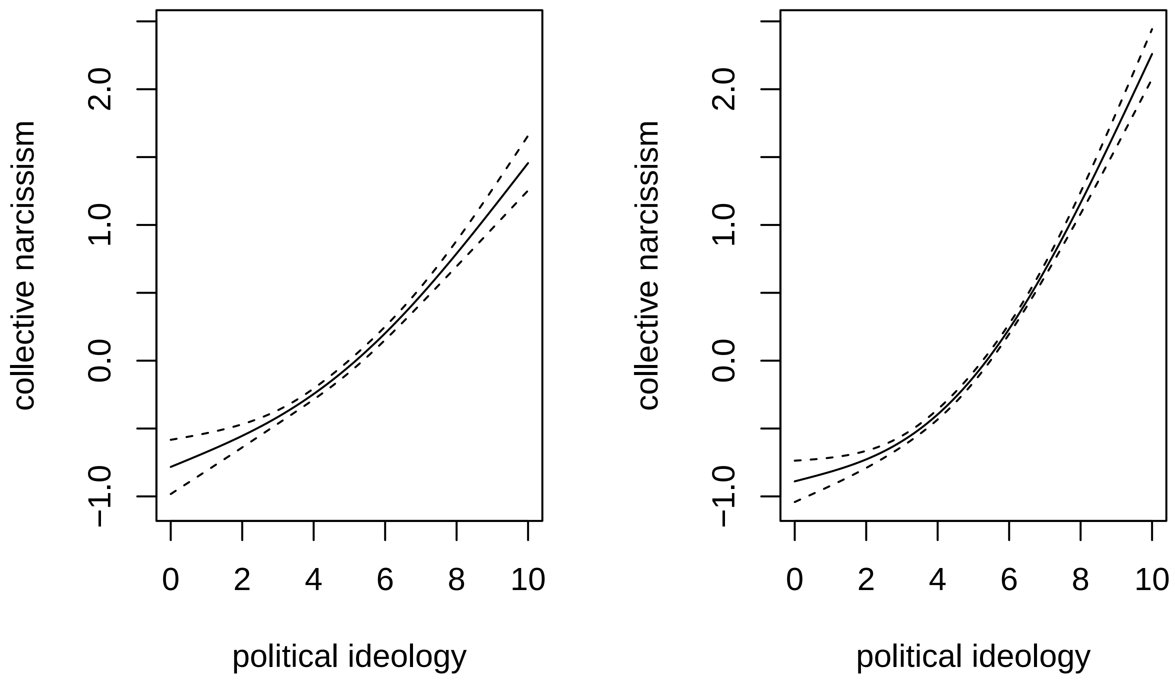

Supplement: Supplementary File 1. — Supplemental Material—Data analyses. [file irsp-37-844-s1.pdf]
